# Targeting the intestinal barrier with traditional Chinese medicine for non-alcoholic fatty liver disease: mechanistic insights and therapeutic perspectives

**DOI:** 10.1186/s13020-025-01311-x

**Published:** 2026-01-09

**Authors:** Xinyue Cheng, Shan Li, Chuntian Huang, Yi Yang, Rong Liu, Shuaibing Cao, Lei Luo, Baoping Lu

**Affiliations:** 1 The Second Clinical Medical College, Henan University of Chinese Medicine, Zhengzhou, 450046 China; 2 Medical College, Henan University of Chinese Medicine, Zhengzhou, 450046 China; 3 Henan Province Hospital of Traditional Chinese Medicine (The Second Affiliated Hospital of Henan University of Traditional Chinese Medicine), Zhengzhou, 450002 China; 4 Henan University of Chinese Medicine, No. 156, Jinshui East Road, Jinshui District, Zhengzhou, 450046 Henan China

**Keywords:** Non-alcoholic fatty liver disease, Traditional Chinese Medicine, Intestinal barrier, Gut microbiota, Bile acid metabolism, Gut-liver axis

## Abstract

Non-alcoholic fatty liver disease (NAFLD) is a chronic liver disease closely associated with metabolic dysregulation, exhibiting a complex and multifactorial pathogenesis. Recent studies demonstrate that impaired intestinal barrier function, intestinal immune barrier dysfunction, gut microbiota dysbiosis, and their associated metabolites collectively promote energy metabolism dysregulation and systemic inflammatory responses via the gut-liver axis. These interconnected processes represent key drivers in the pathogenesis and progression of NAFLD. Notably, Traditional Chinese Medicine (TCM) offers a promising therapeutic approach for NAFLD by holistically modulating intestinal barrier function, immune responses, and gut microbiota composition via multiple targets and pathways, thereby ameliorating gut dysbiosis and its downstream metabolic and inflammatory sequelae. This review systematically synthesizes the established relationship between NAFLD and intestinal barrier dysfunction. It critically evaluates current research progress on TCM interventions—including single herbs, bioactive constituents, and compound formulas—that target the intestinal barrier for NAFLD management. Key mechanistic insights into TCM efficacy are summarized, focusing on the repair of the intestinal mucosal barrier, modulation of bile acid metabolism and gut microbiota composition, and enhancement of intestinal immune barrier function. Future research should prioritize elucidating the specific molecular mechanisms underpinning TCM-mediated intestinal barrier regulation and strengthening clinical validation, ultimately advancing the scientific foundation for TCM-based NAFLD therapeutics.

**Graphical Abstract:**

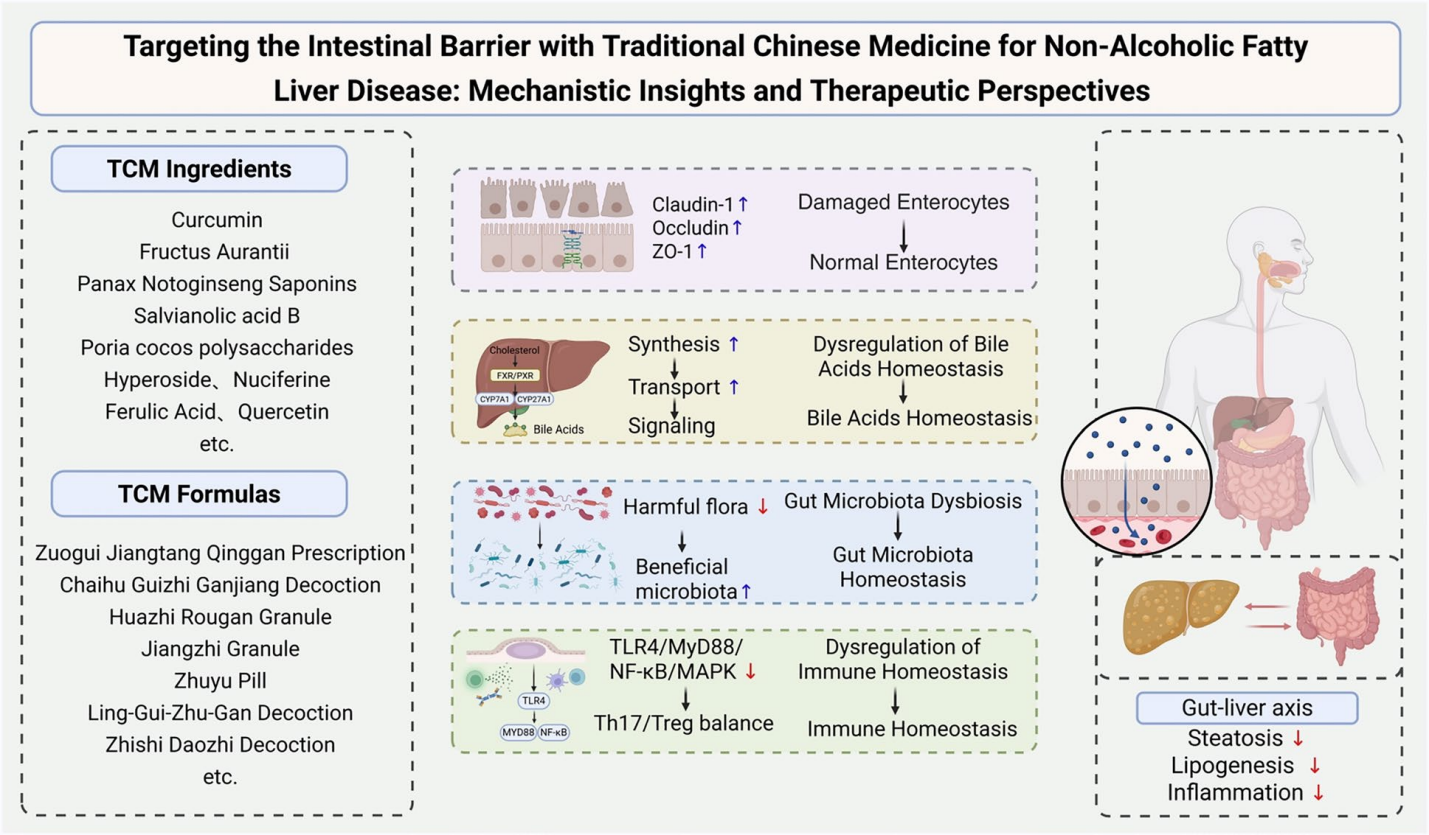

## Highlights

- Links NAFLD pathogenesis to intestinal barrier dysfunction, gut microbiota dysbiosis, and gut-liver axis dysregulation.
- Summarizes TCM interventions (single herbs, bioactives, formulas) for NAFLD via intestinal barrier targeting.
- Clarifies TCM mechanisms: repairing mucosal barrier, regulating bile acid/microbiota, enhancing intestinal immunity.
- Confirm the potential of TCM in managing NAFLD by regulating the gut-liver axis.

## Introduction

Non-alcoholic fatty liver disease (NAFLD), defined histologically by macrovesicular steatosis of hepatocytes, represents a globally prevalent chronic liver condition. Its rapidly rising prevalence has established NAFLD as a critical public health burden with significant worldwide implications [1]. In April 2020, an international consensus panel endorsed the renaming of NAFLD to Metabolism-associated fatty liver disease (MAFLD). This updated nomenclature more accurately reflects the disease's etiology and pathogenesis, emphasizing its intrinsic association with underlying metabolic conditions including obesity, type 2 diabetes, and hypertension [2]. The disease spectrum of NAFLD encompasses a continuum from simple steatosis to nonalcoholic steatohepatitis (NASH), with potential progression to hepatic fibrosis, cirrhosis, and hepatocellular carcinoma [3]. Although the pathogenesis of NAFLD remains incompletely understood, current evidence identifies insulin resistance, oxidative stress, chronic inflammation, and intestinal barrier dysfunction as pivotal drivers of disease initiation and progression [4].

In recent years, the association between intestinal barrier dysfunction and NAFLD has garnered significant attention. The intestinal barrier constitutes a highly dynamic system, encompassing four principal components: the mechanical, chemical, biological, and immune barriers. Collectively, these components safeguard the gastrointestinal tract against invasion by pathogenic microorganisms, endotoxins, and antigens, thereby contributing to the maintenance of systemic homeostasis [5]. Impairment of the intestinal barrier facilitates the translocation of detrimental substances, including endotoxins and bacterial metabolites, into the systemic circulation. These substances subsequently reach the liver via the portal venous system, instigating hepatic inflammatory responses and insulin resistance. Critically, intestinal barrier dysfunction is implicated in the pathogenesis of a spectrum of systemic metabolic and autoimmune disorders, encompassing obesity, type 1 diabetes mellitus (T1DM), NAFLD, neurodegenerative diseases, cardiovascular diseases (CVD), and inflammatory bowel disease (IBD) [6–8]. A bidirectional communication network, termed the "gut-liver axis", functionally interconnects the intestine and the liver through mutual interactions. Therapeutic targeting of this axis to modulate intestinal barrier function represents a promising strategy for the management of NAFLD.

Traditional Chinese Medicine (TCM), a cornerstone of Chinese medical heritage, employs a holistic therapeutic strategy guided by syndrome differentiation. Accumulating evidence indicates its significant potential in ameliorating intestinal barrier dysfunction and NAFLD. Mechanistic studies reveal that TCM interventions restore gut microbiota homeostasis, enhance intestinal mucosal barrier integrity, and regulate immune barrier function via multi-target mechanisms [9]. These actions collectively preserve intestinal barrier competence, attenuate translocation of endotoxins and microbial metabolites, and ultimately mitigate NAFLD pathogenesis. Substantial research efforts in recent years have investigated the therapeutic potential of TCM – encompassing bioactive monomers, active constituents, and complex formulations – in modulating intestinal barrier function and ameliorating NAFLD. While these investigations have yielded significant progress, the precise molecular mechanisms underpinning these effects remain incompletely characterized, necessitating further mechanistic exploration. This review systematically examines the pathophysiological link between intestinal barrier impairment and NAFLD pathogenesis, and synthesizes contemporary advances in understanding the cellular and molecular mechanisms through which TCM interventions target intestinal barrier dysfunction in NAFLD.

### Structural components and functional dynamics of the intestinal barrier system

**Fig. 1:**
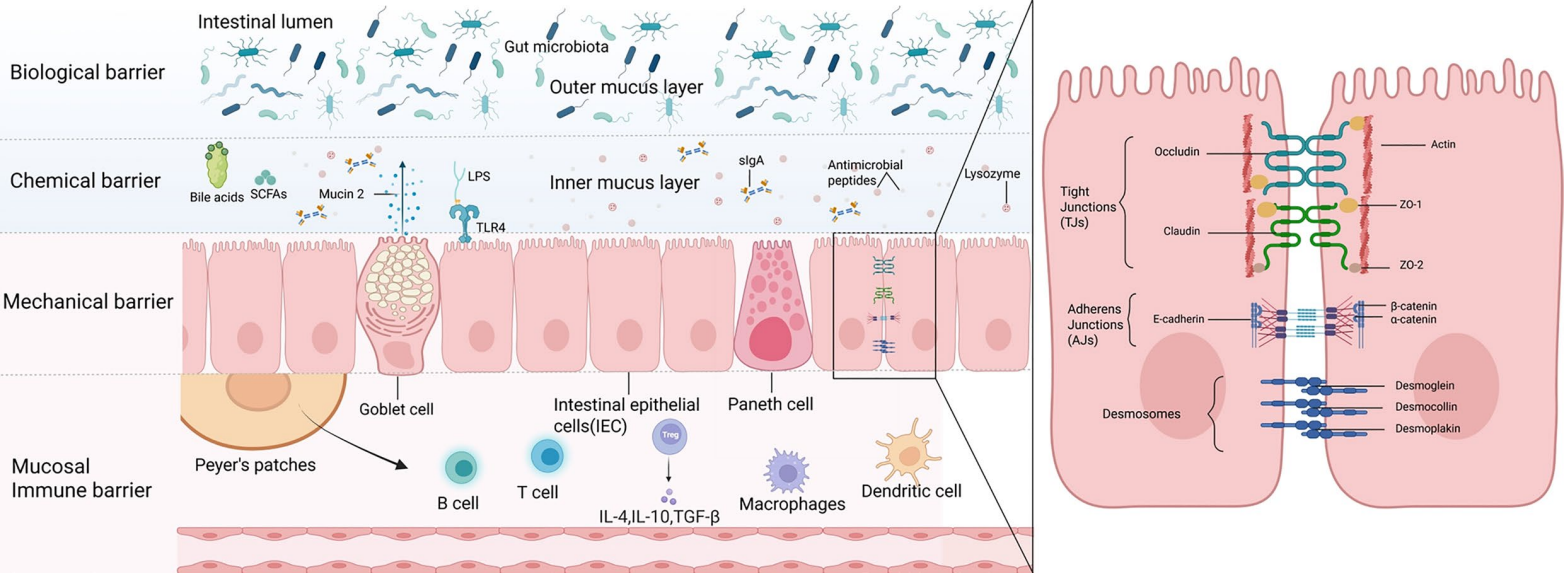
Schematic representation of the multilayered intestinal barrier. The intestinal barrier integrates four synergistic defense mechanisms to maintain homeostasis. A mechanical barrier of intestinal epithelial cells, interconnected by tight junctions and other adhesion structures, forms a selective physical seal. This is reinforced by a chemical barrier comprising bile acids, short-chain fatty acids (SCFAs), antimicrobial peptides, and lysozyme, which collectively inhibit pathogenic microorganisms. The biological (microbiota) barrier, constituted by the commensal gut flora, provides colonization resistance and is regulated by the overlying mucus layer and secretory immunoglobulin A (sIgA). Underlying these layers, an immune barrier, featuring immune cells such as B cells, T cells, macrophages, and dendritic cells within organized structures like Peyer's patches, orchestrates targeted responses via cytokines to eliminate pathogens while preserving tolerance to benign antigens. This figure illustrates the four core components of the intestinal barrier—mechanical, chemical, biological, and immune—and their synergistic action, collectively preventing harmful substances in the gut from entering the bloodstream and maintaining systemic homeostasis

The intestinal barrier system constitutes a primary defense mechanism against luminal pathogens and noxious compounds. This integrated entity comprises four interdependent components: the mechanical, chemical, biological, and immune barriers. Through coordinated interactions, these elements collectively preserve intestinal microenvironmental homeostasis and organismal health (Fig. 1).

### Structural and regulatory dynamics of the mechanical barrier: epithelial junctional complexes in intestinal permeability control

The intestinal mechanical barrier constitutes the primary physical defense of the gastrointestinal tract, comprising the intestinal mucosal epithelium, intercellular junctional complexes, and the overlying mucus layer. The intestinal mucosal epithelium consists of a single layer of columnar epithelial cells, including absorptive enterocytes responsible for nutrient uptake, goblet cells that secrete the gel-forming glycoprotein mucin 2 (MUC2) as the primary constituent of the mucus layer, and Paneth cells residing in the crypt bases which secrete antimicrobial peptides (e.g., defensins, cryptdins) and lysozyme contributing to innate immune defense, and enteroendocrine cells that release hormones regulating intestinal function [10]. The mucus layer, primarily composed of MUC2 secreted by goblet cells, exhibits a stratified structure: its outer layer is loose, non-adherent, harbors a dense commensal microbiota, and is readily cleared by intestinal peristalsis; its inner layer is dense, firmly adherent, forms a viscous, gel-like network directly overlying the epithelial surface, minimizes mechanical damage during luminal content transit, and prevents direct contact between luminal pathogens and the epithelium [11]. Epithelial junctional complexes, located at the apical-lateral borders of adjacent cells, maintain epithelial integrity and regulate paracellular permeability; these complexes primarily include tight junctions (TJs), forming the most apical semi-permeable seal regulating ion and solute passage, adherens junctions (AJs) located subjacent to TJs providing strong mechanical adhesion via cadherin-catenin complexes, and desmosomes acting as anchoring spots providing robust intercellular adhesion along the lateral membranes below AJs.By forming semi-permeable seals within intercellular spaces, TJs selectively permit the paracellular passage of water, ions, and small nutrient molecules while excluding luminal pathogens, toxins, and macromolecular substances from entering underlying tissues. These junctional complexes constitute the fundamental structural basis of the intestinal mechanical barrier and are indispensable for maintaining intestinal epithelial integrity and homeostatic function [12]. Intestinal epithelial cells establish a continuous monolayer through TJs, which comprise transmembrane proteins (e.g., occludin), claudin family proteins (e.g., claudin-1), and cytoplasmic scaffolding proteins (e.g., ZO-1, ZO-2, ZO-3).[13]. Tight junction proteins are interwoven with each other, effectively restricting paracellular transport of substances and preventing harmful substances such as bacteria and endotoxins from entering the bloodstream from the intestinal lumen, which is crucial for maintaining the integrity of the intestinal barrier [14]. Positioned subjacent to TJs, AJs consist principally of the transmembrane glycoprotein E-cadherin and the adaptor protein β-catenin. The extracellular domain of E-cadherin mediates calcium-dependent homophilic adhesion through trans-interactions with E-cadherin molecules on adjacent cells [15]. Intracellularly, E-cadherin binds β-catenin, which associates with α-catenin to establish a molecular bridge anchoring the adhesion complex to the actin cytoskeleton. This structural linkage not only stabilizes intercellular adhesion but also regulates fundamental cellular processes including proliferation, differentiation, and migration [16]. Compromise of adherens junctions impairs tight junction functionality, resulting in disruption of the intestinal mechanical barrier. Desmosomes represent structurally robust intercellular junctions composed primarily of cadherin-family proteins (desmogleins, desmocollins) and plaque proteins (desmoplakins). These components interface with intermediate filaments to assemble a stress-resistant scaffold that confers tensile resistance to intestinal epithelia, enabling cellular adaptation to luminal mechanical stresses [17]. Furthermore, during lumen-distending forces or peristaltic contractions, desmosomes prevent shear-induced intercellular dissociation, thereby preserving epithelial barrier continuity.

Intestinal barrier permeability serves as a key functional metric of the intestinal mechanical barrier, quantifying its capacity to regulate paracellular flux. Under physiological conditions, this barrier exhibits selective permeability: facilitating transjunctional passage of essential nutrients (e.g., glucose, amino acids, short-chain fatty acids, electrolytes) while restricting translocation of potentially pathogenic macromolecules including bacterial lipopolysaccharide (LPS), endotoxins, and immunoglobulins [18]. Intestinal barrier permeability undergoes precise physiological regulation through neuroendocrine-immunological modulators. Luminal and systemic neurotransmitters, hormones, and cytokines bind cognate receptors on intestinal epithelial cells, activating intracellular signaling cascades that increase paracellular permeability. These pathways induce downregulation of tight junction protein expression, their mislocalization, and actin cytoskeleton reorganization—collectively compromising barrier integrity [19]. Furthermore, oxidative stress and endoplasmic reticulum (ER) stress induce intestinal epithelial cell damage and disrupt junctional complex architecture, culminating in elevated intestinal barrier permeability [20, 21]. Clinical and experimental evidence indicates that during intestinal injury or pathological conditions in NAFLD, compromised barrier function permits translocation of luminal pathogens and pathogen-associated molecular patterns (PAMPs; e.g., bacteria, endotoxins) into systemic circulation. This microbial translocation activates innate immune responses, triggering systemic inflammatory cascades [4].

### Biochemical Mediators of Intestinal Chemical Barrier Function: Bile Acids

The intestinal chemical barrier constitutes a critical defense mechanism against xenobiotic incursion, comprising bioactive agents including bile acids (BAs), gastric acid, digestive enzymes, mucins, and antimicrobial peptides. These molecular components collectively preserve intestinal microenvironmental homeostasis and safeguard systemic health [22]. BAs constitute a class of cholesterol-derived amphipathic molecules synthesized in the liver as primary BAs. Following hepatobiliary excretion into the intestinal lumen, BAs facilitate lipid emulsification, digestion, and absorption. Beyond their digestive functions, BAs serve as critical signaling molecules regulating cholesterol homeostasis, energy metabolism, and nutrient assimilation. Gut microbiota biotransform primary BAs into secondary BAs through enzymatic modifications, predominantly via deconjugation and dehydroxylation. Approximately 95% of intestinal BAs undergo efficient ileal reabsorption and return to the liver via portal venous circulation—a tightly regulated process termed enterohepatic circulation [23]. Peroxisome proliferator-activated receptor α (PPARα) and Farnesoid X receptor (FXR) orchestrate hepatic lipid homeostasis through transcriptional regulation of metabolic genes. These nuclear receptors coordinately modulate lipid utilization, BAs biosynthesis, and transport pathways [24]. FXR activation suppresses de novo lipogenesis by downregulating sterol regulatory element-binding protein 1c (SREBP-1c), a master lipogenic transcription factor. Concordantly, FXR-deficient mice exhibit hepatic steatosis with elevated intrahepatic triglyceride (TG) accumulation and hyperlipidemia characterized by increased serum TG and cholesterol levels [25]. Elevated BAs concentrations activate FXR, inducing expression of small heterodimer partner (SHP). The SHP-receptor complex interacts with hepatocyte nuclear factor 4α (HNF4α) or liver receptor homolog-1 (LRH-1), competitively inhibiting their binding to promoter regions of CYP7A1 and CYP8B1. This transcriptional repression downregulates cholesterol 7α-hydroxylase and 12α-hydroxylase expression, ultimately suppressing de novo BAs synthesis and restoring bile acid homeostasis through negative feedback regulation [26]. CYP7A1 serves as the rate-limiting enzyme in the classical BAs biosynthetic pathway, catalyzing the 7α-hydroxylation of cholesterol to form 7α-hydroxycholesterol. Concurrently, CYP27A1 mediates the alternative pathway through 27-hydroxylation of cholesterol, generating 27-hydroxycholesterol and facilitating cholesterol catabolism [27]. Beyond their biosynthetic functions, BAs regulate systemic energy expenditure, glucose homeostasis, and immunomodulation via receptor activation: nuclear vitamin D receptor (VDR) and membrane-bound G protein-coupled bile acid receptor 1 (GPBAR1/TGR5). These signaling pathways critically modulate innate immunity and metabolic homeostasis [28, 29]. BAs exert significant regulatory effects on the intestinal microbiota. An imbalance in bile acid levels, particularly abnormally elevated concentrations, can inhibit the growth of commensal bacteria while promoting the proliferation of potentially pathogenic bacteria. This disruption alters the composition and functional capacity of the gut microbiota, ultimately leading to intestinal dysbiosis [30]. Furthermore, BAs metabolites inhibit FXR signaling. This inhibition counteracts aspirin-induced intestinal microbiota dysbiosis and barrier damage, while concurrently promoting Wnt signaling and facilitating intestinal epithelial repair [31]. Circulating via the enterohepatic circulation, BAs play a key role in systemic lipid metabolism regulation. They modulate the expression of hepatic glucose and lipid metabolism-related genes primarily through FXR and GPBAR1, thereby exerting indirect effects on lipid metabolism and overall energy homeostasis [32].

### Intestinal biological barrier: role of the gut microbiota

The intestinal biological barrier, a critical component of the gut barrier system, is principally constituted by a diverse commensal microbiota [33]. This microbial community encompasses bacteria, fungi, and viruses, with bacteria representing the dominant domain. At the phylum level, Bacteroidetes and Firmicutes predominate, followed by Actinobacteria and Proteobacteria [34]. These microorganisms contribute to pathogen resistance, maintenance of intestinal epithelial function, and regulation of gut immunity [35]. Key commensals including Bifidobacterium and Lactobacillus spp. establish extensive colonization within the gastrointestinal tract and play a vital role in intestinal homeostasis [36].

The gut microbiota maintains symbiosis with the intestinal mucosa, underpinning critical metabolic, immune, and protective functions in the healthy host through two primary mechanisms. Firstly, commensals utilize nutrients derived from dietary components and exfoliated epithelial cells. This metabolic activity competitively excludes pathogens from essential nutritional resources and mucosal adhesion sites, thereby preventing colonization by harmful bacteria [37]. Concurrently, microbiota-derived antimicrobial compounds—including bacteriocins and short-chain fatty acids (SCFAs)—selectively inhibit pathogens. SCFAs (principally acetate, propionate, and butyrate produced through dietary fiber fermentation) lower luminal pH, creating an inhospitable environment for pathogens. Furthermore, SCFAs promote microbiota equilibrium and enhance intestinal barrier integrity by stimulating mucin production and reinforcing tight junctions [38]. Notably, butyrate serves as the primary energy source for intestinal epithelial cells (IECs), fueling their proliferation, differentiation, and repair. This metabolic support is crucial for maintaining mucosal integrity [39]. Additionally, substantial evidence indicates that beneficial gut microbiota contribute significantly to intestinal barrier integrity and gastrointestinal structure. This is achieved through the restoration of tight junction protein complexes and the upregulation of mucin gene expression [40]. For instance, early administration of Bifidobacterium bifidum BD-1 elevates Claudin-1 and Occludin expression, preserving the structural integrity of the intestinal epithelium [41]. During intestinal immune system development, the gut microbiota and their metabolites (including SCFAs, tryptophan, and BAs) play pivotal immunomodulatory roles. They promote the differentiation and function of immunoregulatory cells and modulate the activity of gut-resident immune cells, such as macrophages and dendritic cells. These interactions facilitate the activation of T and B lymphocytes, enhancing specific immune responses for effective defense against pathogen invasion. Moreover, microbiota metabolites inhibit inflammatory cytokine secretion by innate immune cells, including natural killer (NK) cells and neutrophils, thereby reducing inflammatory responses and maintaining intestinal and systemic immune homeostasis [42].

### Mucosal immune system: provider of intestinal immune barrier function

The intestinal immune barrier—comprising cellular and humoral components—constitutes a critical element of the systemic immune system. Studies confirm that the gut harbors the body's largest and most diverse array of immune compartments and cells [43]. The cellular immune barrier integrates gut-associated lymphoid tissue (GALT), immune cells, and immune molecules, forming an essential defense layer.GALT represents the most extensive lymphoid tissue network in humans, encompassing Peyer’s patches, mesenteric lymph nodes, and isolated lymphoid follicles. Based on anatomical distribution, resident lymphocytes are classified as intraepithelial lymphocytes (IELs) or lamina propria lymphocytes (LPLs) [44]. This system generates diverse immune cells (T lymphocytes, B lymphocytes, macrophages, dendritic cells) that surveil the intestinal lumen, recognize pathogens and antigens, and initiate immune responses [45, 46]. These cells execute immune defense, surveillance, and regulation through cytokine secretion (e.g., IL-1β, IL-2, IL-4, IL-10) [47]. Intestinal epithelial cells express Toll-like receptors (TLRs), key pattern recognition receptors of innate immunity. TLRs are predominantly localized on epithelial cells, macrophages, and dendritic cells, where they detect pathogen-associated molecular patterns (PAMPs) or damage-associated molecular patterns (DAMPs) [48]. TLR engagement activates nuclear factor-κB (NF-κB) and mitogen-activated protein kinase (MAPK) signaling pathways, driving macrophage activation and upregulating pro-inflammatory mediators—including tumor necrosis factor-α (TNF-α) and interleukin-6 (IL-6) [49].

Secretory immunoglobulin A (SIgA) constitutes the predominant immunoglobulin within the intestinal mucosal humoral immune barrier. LPLs primarily comprise IgA⁺ plasma cells and CD4⁺ Th2 cells. IgA⁺ plasma cells secrete abundant SIgA, while CD4⁺ Th2 cells facilitate the differentiation and maturation of IgA⁺ B cells into IgA⁺ plasma cells [50]. SIgA cooperates with the mucus layer to directly bind commensal bacteria, inhibiting their adhesion to and invasion of intestinal epithelial cells. Furthermore, it promotes clearance of bacteria penetrating the mucosal barrier, thereby preventing barrier compromise. SIgA additionally neutralizes bacterial toxins and viruses, fulfilling critical immune-protective functions. Concurrently, intestinal immune cells contribute to microenvironmental stability through modulation of gut microbiota composition [51].

### Intestinal barrier dysfunction in NAFLD

#### Intestinal structural barrier impairment and consequent inflammation in NAFLD

**Fig. 2:**
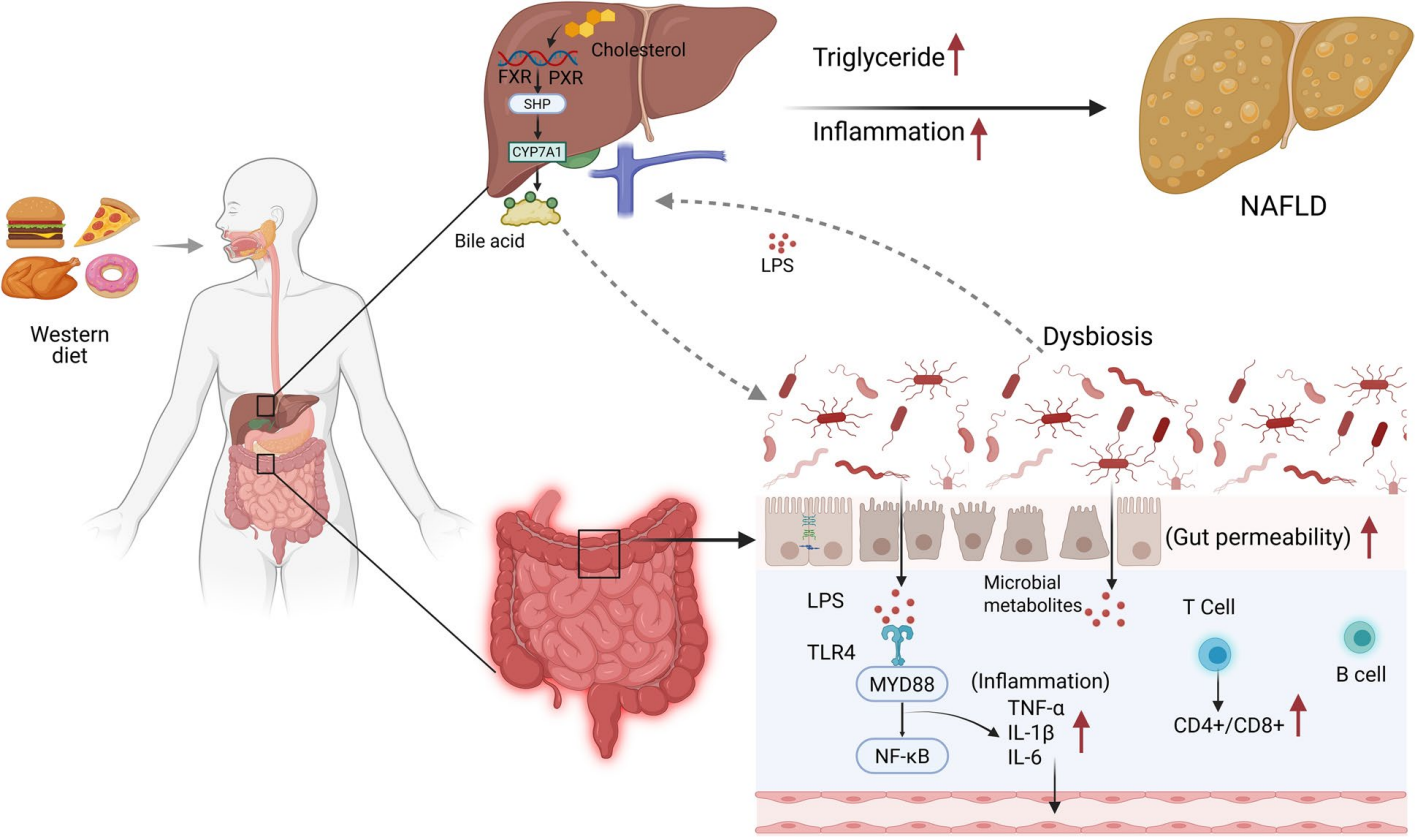
Mechanisms by which intestinal barrier impairment drives the pathogenesis of NAFLD via the gut-liver axis. In this figure illustrates the core pathway through which NAFLD develops via the gut-liver axis: long-term consumption of a Western diet disrupts hepatic cholesterol and bile acid metabolism (involving key regulators such as FXR and PXR). Simultaneously, it induces gut microbiota dysbiosis, which compromises the intestinal barrier and increases intestinal permeability ("leaky gut"), allowing bacterial toxins like LPS to enter the portal circulation. Upon reaching the liver, LPS activates the TLR4 receptor on hepatic immune cells, triggering the key MYD88-NF‑κB inflammatory signaling pathway and promoting the release of large quantities of pro‑inflammatory cytokines (e.g., TNF‑α, IL‑6). This local inflammation, combined with systemic immune activation (involving T cells, B cells, etc.) and elevated hepatic triglyceride levels, creates a sustained vicious cycle that ultimately drives the progression of NAFLD from simple steatosis to inflammatory and fibrotic stages. This figure reveals the pathological process by which Western diet induces NAFLD. Specifically, Western diet impairs the intestinal barrier, disrupts bile acid metabolism and gut microbiota, and subsequently triggers hepatic inflammation and lipid accumulation via the TLR4-MyD88-NF-κB pathway, ultimately leading to NAFLD

Diet-induced elevation of systemic LPS levels, termed metabolic endotoxemia, is a key driver in the pathogenesis of chronic metabolic disorders, including obesity, type 2 diabetes, and NAFLD. The intestinal epithelium normally serves as a selective barrier restricting LPS translocation [52]. However, consumption of a Western diet (characterized by high fat, sugar, saturated fatty acids, and cholesterol) not only increases the risk of NAFLD but also induces structural alterations within the intestinal epithelium [53] (Fig. 2). These alterations compromise barrier integrity, increase intestinal permeability, and facilitate the systemic entry of luminal toxins, including LPS. Subsequent hepatic Toll-like receptor 4 (TLR4) activation by translocated LPS stimulates immune cells, induces pro-inflammatory cytokine production, and drives progression from steatosis to fibrosis, accelerating NAFLD pathogenesis [54–56]. Tight junction proteins are critical for maintaining the intestinal epithelial barrier. Studies demonstrate that expression levels of the intestinal tight junction proteins Occludin, Claudin-1, and ZO-1 are significantly reduced in NAFLD patients compared to healthy subjects [57]. Consistent with this, Anika et al. [58] reported significantly elevated plasma endotoxin and inflammatory marker levels in NAFLD patients, which increased with hepatic steatosis severity. Furthermore, in LPS-induced NASH mouse models, Occludin and ZO-1 protein expression levels in intestinal tissues were markedly reduced compared to normal controls, accompanied by widened intercellular gaps, increased intestinal permeability, and a consequent weakening of the intestinal barrier's defensive capacity [59]. Furthermore, the study identified that NAFLD-associated metabolic disorder products, free fatty acids (FFAs), and D-lactate activate the intracellular RhoA/ROCK signaling pathway [60]. This activation alters phosphorylation levels of actin-binding proteins, triggering cytoskeletal depolymerization and remodeling. These structural changes impair intestinal epithelial barrier function, exacerbating hepatic inflammation and steatosis. NAFLD is frequently characterized by significant oxidative stress. Excessive reactive oxygen species (ROS) disrupt cellular redox homeostasis and directly induce oxidative modifications (e.g., carbonylation, disulfide bond disruption) in tight junction proteins, compromising gastrointestinal barrier integrity and increasing intestinal permeability. Additionally, ROS function as signaling mediators, activating intracellular oxidative stress pathways such as NF-κB. This leads to the release of pro-inflammatory cytokines, including TNF-α, IL-1β, and IL-6. Collectively, these mechanisms disrupt the intestinal epithelial barrier, enhancing permeability and facilitating the translocation of enteric toxins (e.g., endotoxin) into the portal circulation. Subsequent hepatic exposure aggravates liver injury [61].

### Bile acids dysregulation

Under physiological conditions, BAs are synthesized from cholesterol in the liver via a series of enzymatic reactions. Following their digestive and regulatory functions within the intestine, BAs undergo gut microbiota-mediated transformations (e.g., deconjugation, dehydroxylation) and are subsequently reabsorbed into the portal venous circulation, completing the enterohepatic cycle [62]. BAs dysregulation can directly or indirectly impair the intestinal epithelial barrier. Elevated concentrations of hydrophobic BAs incorporate into the lipid bilayer of intestinal epithelial cell membranes. This incorporation damages epithelial cells, alters membrane fluidity and stability, disrupts the normal expression and distribution of TJs proteins (e.g., Occludin, Claudin-1), and compromises TJs integrity [63, 64]. Corroborating this, studies demonstrate reduced expression of intestinal TJs proteins (Occludin, Claudin-1), widened intercellular spaces, and increased intestinal permeability under conditions of BAs imbalance. This enhanced permeability facilitates the translocation of harmful luminal substances, including bacteria and endotoxins, into the portal circulation, thereby exacerbating hepatic inflammation and steatosis [65]. Furthermore, studies in NAFLD patients reveal alterations in the expression and activity of key enzymes governing bile acid synthesis and metabolism. Notably, hepatic CYP7A1 expression is reduced in NAFLD, leading to diminished BA synthesis [66]. Research demonstrates that in high-fat diet (HFD)-induced NAFLD mouse models, hepatic CYP7A1 mRNA and protein expression are significantly diminished. This reduction impairs BAs synthesis, compromises intestinal barrier function, and disrupts lipid digestion and absorption [67]. Conversely, CYP7A1 upregulation effectively remodels BAs metabolism, enhancing BAs synthesis. This intervention reduces hepatic lipid accumulation, suppresses inflammation, alleviates fibrosis, and ameliorates liver injury, thereby mitigating HFD-induced NASH [68].

### Gut microbiota dysbiosis

The gut microbiota plays crucial roles in human energy metabolism. Evidence indicates that intestinal dysbiosis—characterized by altered microbial diversity, shifted community gene expression, and perturbed metabolic pathways—contributes to NAFLD pathogenesis through multiple mechanisms. These include disrupted energy homeostasis, endotoxemia-triggered inflammation, and aberrant bile acid metabolism [69, 70]. Notably, studies reveal a significant reduction in Bifidobacterium abundance in NAFLD patients compared to healthy controls. This depletion diminishes the production of SCFAs, compromising energy supply and metabolic regulation in intestinal epithelial cells. Consequently, these disturbances propagate to the liver, driving hepatic energy metabolism dysregulation and lipid accumulation [71]. Studies on the gut microbiota characteristics of non-obese NAFLD patients have shown that nine bacterial genera, including Fusobacterium, Lachnospira, and Klebsiella, are associated with abnormal liver enzymes and glucose-lipid metabolic disorders. Pharmacological intervention with ursodeoxycholic acid (UDCA) increased the abundance of beneficial genera such as Alistipes, Holdemanella, and Christensenella. This microbial shift restored microbiota balance, concurrently reducing hepatic transaminase levels and liver fat content in NAFLD patients [72]. Comparative analyses further demonstrate significant gut dysbiosis in NAFLD and NASH. Huang et al. utilizing 16S rRNA gene sequencing, reported markedly reduced microbial diversity in patients versus healthy controls, accompanied by elevated abundances of potentially detrimental phyla such as Fusobacteria and Bacteroidetes [73]. Complementing this, a multi-methodological study (employing MALDI-TOF MS of bacterial cultures, qPCR, and 16S rRNA gene NGS) compared non-fibrotic NAFLD patients to controls. Results revealed enrichment of Collinsella (genus) and Firmicutes (phylum) in controls, while NAFLD patients exhibited a significantly higher abundance of Enterococcus (genus) [74]. Furthermore, a systematic review and meta-analysis established gut microbiota dysbiosis as a key driver of NAFLD progression. This dysbiosis is characterized by depletion of anti-inflammatory taxa (Ruminococcaceae family, Coprococcus genus) and enrichment of pro-inflammatory genera (Fusobacterium, Escherichia) in NAFLD patients [75]. Mechanistic evidence from murine models confirms this pathogenic role. In high-fat/high-cholesterol (HFHC) diet-fed mice, depletion of Bifidobacterium and Bacteroides induced dysbiosis that promoted NAFLD development and remarkably accelerated progression to hepatocellular carcinoma [76]. Similarly, HFD-fed obese mice showed gut microbiota alterations that increased intestinal permeability by downregulating tight junction proteins (ZO-1, Occludin), elevated plasma LPS and pro-inflammatory cytokine levels, and triggered systemic endotoxemia and inflammatory cascades [77]. The gut microbiota critically regulates BAs enterohepatic circulation and metabolic transformation. In NAFLD, gut dysbiosis disrupts physiological BAs metabolism and enterohepatic cycling, impairing intestinal BAs reabsorption and compromising BAs-mediated regulation of hepatic lipid homeostasis [78]. Mechanistically, disulfiram administration in NASH model mice inhibits Clostridium species-mediated 7α-dehydroxylation activity, thereby suppressing secondary BAs biosynthesis. This reduction activates hepatic FXR signaling, ultimately ameliorating NASH pathology. Notably, this protective effect is recapitulated in clinical trials, confirming therapeutic efficacy [79].

### Dysregulation of mucosal immune homeostasis

Intestinal immune cells critically orchestrate microbial community homeostasis and reinforce epithelial barrier integrity through regulated immune-epithelial crosstalk. Concurrently, intestinal microenvironmental stability is essential for maintaining multilayered barrier function. Dysregulated activation of mucosal immunity represents a primary driver of mucosal inflammation in both gastrointestinal inflammatory diseases and metabolic disorders [80]. This immune imbalance promotes hepatic pathology through the gut-liver axis: aberrant release of cytokines and inflammatory mediators by intestinal immune cells exerts direct hepatotoxic effects via portal circulation, inducing de novo liver injury or exacerbating pre-existing lesions, ultimately compromising hepatic physiological function. Although the mechanistic contributions of intestinal immune dyshomeostasis to NAFLD pathogenesis require further elucidation, consistent evidence from human studies and animal models demonstrates immune cell infiltration indicative of mucosal inflammation and elevated pro-inflammatory cytokine profiles (e.g., TNF-α, IL-6, IL-1β) during both NAFLD and NASH progression [81, 82]. Studies demonstrate that expanded intestinal B-cell populations in both human and murine NASH models promote metabolic T-cell activation, thereby exacerbating disease progression. These B cells further activate the IgA-FcR signaling axis through secreted IgA, which specifically triggers CD11b⁺CCR2⁺F4/80⁺CD11c⁻FCGR1⁺ hepatic myeloid cells to drive liver fibrosis — a process independent of gut microbiota involvement [83]. Complementary findings by Su et al. in HFD-induced NAFLD rats revealed elevated CD4⁺/CD8⁺ T-cell ratios in peripheral blood mononuclear cells (PBMCs) and Peyer’s patches (PPs), concomitant with increased intestinal secretory SIgA production. Concurrently, multiple profibrotic stimuli — including endotoxins and bacterial translocation from gut hyperpermeability, hepatocyte damage-associated factors, and gut microbiota-derived metabolites — collectively induce macrophage activation [84]. Critically, polarization of macrophages toward a pro-inflammatory phenotype mediates steatosis progression, hepatic inflammation, and fibrogenesis in NAFLD [85].

Hepatocyte-derived DAMPs, saturated fatty acids, and gut-derived endotoxins act as ligands for TLRs. TLR activation induces macrophage-driven inflammatory cascades, thereby propagating NAFLD pathogenesis [86]. Substantiating this mechanism, murine studies with macrophage-specific deletion of myeloid differentiation primary response 88 (MyD88) demonstrated that inhibition of TLR/NF-κB-mediated macrophage inflammation significantly attenuates hepatic lipid accumulation and injury in NAFLD models [87]. Collectively, these findings implicate TLR-dependent mucosal immunity as a critical effector of intestinal barrier dysfunction in NAFLD. Nevertheless, therapeutic targeting of intestinal immune homeostasis requires further investigation to define its role in NAFLD and broader metabolic disorders.

### Mechanisms of traditional Chinese medicine in modulating intestinal barrier homeostasis for NAFLD management

**Fig. 3:**
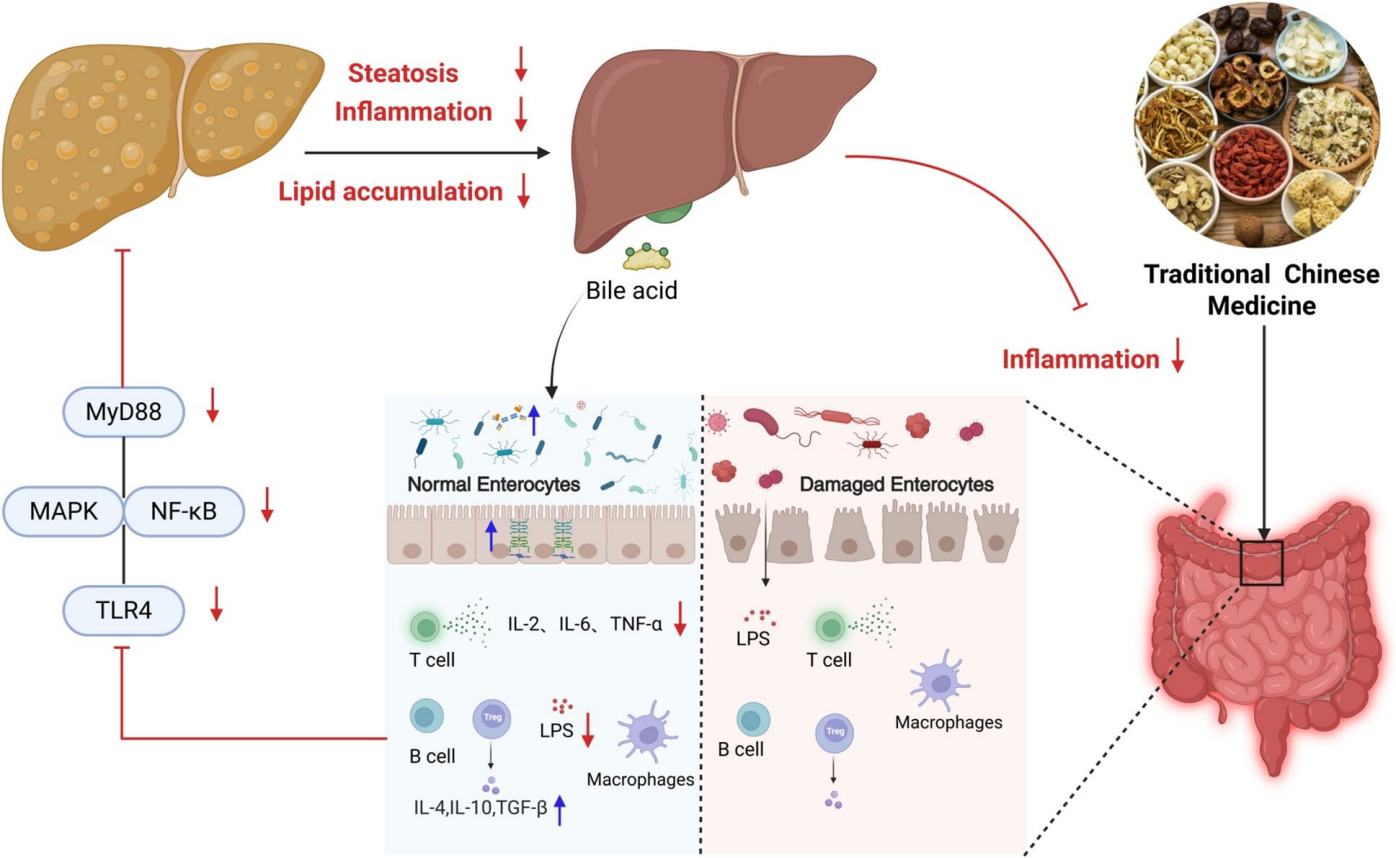
Integrated mechanisms by which TCM treats NAFLD through multi‑target regulation of the gut-liver axis. The pathogenesis of NAFLD begins with hepatic steatosis, inflammation, and lipid accumulation, a process closely associated with intestinal barrier impairment. Dysbiotic gut microbiota can damage the intestinal barrier, leading to translocation of endotoxin (LPS) and activation of the TLR4-MyD88-MAPK/NF-κB inflammatory pathway, accompanied by disrupted bile acid metabolism. The translocated LPS further activates intestinal immune cells (e.g., T cells, macrophages), disrupting the balance between pro-inflammatory cytokines (IL-6, TNF-α) and anti-inflammatory cytokines (IL-10, TGF-β). TCM exerts multi-pathway synergistic interventions: at the intestinal level, it directly modulates the composition and restores the balance of gut microbiota, thereby repairing the intestinal epithelial barrier and reducing LPS translocation; at the hepatic level, it alleviates steatosis, suppresses inflammation and lipid accumulation, and regulates bile acid metabolism; simultaneously, it holistically modulates intestinal and systemic immune responses. Through these coordinated actions, TCM mitigates inflammation in both the gut and the liver, ultimately achieving integrated treatment of NAFLD. (Red ↓: indicate decrease; Blue ↑: indicate increase)

Intestinal barrier dysfunction critically contributes to the progression of NAFLD. Substantial evidence confirms the significant therapeutic efficacy of TCM in NAFLD management. Preclinical and clinical studies demonstrate that TCM interventions—encompassing compound formulations, single-herb extracts, and ingredients—mitigate NAFLD pathogenesis through multifaceted mechanisms including restoration of intestinal mechanical barrier integrity, attenuation of chronic inflammatory responses, modulation of bile acid homeostasis, regulation of gut microbiota composition, and normalization of immune function. Collectively, these actions retard NAFLD onset and disease progression as schematically summarized in Fig. 3. This review synthesizes research on TCM bioactive compounds, ingredients, monomers, and complex formulations, highlighting their molecular mechanisms targeting intestinal barrier dysfunction for NAFLD intervention. Specifically, TCM confers intestinal barrier protection by repairing epithelial tight junctions, suppressing pro-inflammatory cytokine cascades, reestablishing bile acid enterohepatic circulation, restoring microbial eubiosis, and recalibrating mucosal immunity. These multi-targeted modulatory effects underscore TCM's significant therapeutic value in clinical NAFLD management. Consequently, the integrated mechanistic actions establish TCM-mediated intestinal barrier modulation as a viable therapeutic approach for NAFLD and associated metabolic disorders (Fig. 3).

### Restoration of intestinal mechanical barrier and attenuation of chronic inflammation

TCM ingredients, compounds, and compound formulas are capable of regulating the expression of intestinal tight junction proteins and their associated signaling pathways. Accumulating empirical evidence has demonstrated that TCM interventions enhance intestinal barrier integrity predominantly by upregulating the expression of key intestinal epithelial tight junction proteins, such as ZO-1 and occludin. This regulatory effect not only reduces intestinal permeability and inhibits endotoxin translocation but also suppresses pro-inflammatory signaling cascades—with the TLR4/NF-κB pathway being a critical target—thereby mitigating inflammation-induced hepatocellular damage and improving hepatic lipid deposition. By exerting these multi-targeted therapeutic effects, TCM provides a promising gut-liver axis-targeted intervention strategy for NAFLD. A growing body of preclinical and clinical research has consistently confirmed that numerous TCM ingredients, bioactive compounds, and herbal formulas exhibit remarkable efficacy in restoring intestinal barrier function and retarding the progression of NAFLD (Tables 1 and 2).

**Table 1:** Classification, sources, and intestinal barrier-regulating mechanisms of TCM ingredients/compounds in NAFLD management

Mechanism	Ingredients/compounds	TCM (Latin Name)	Medicinal part	Category	Target / medium	Reference
Restore the intestinal mechanical barrier and mitigate chronic inflammatory response	Curcumin	Jianghuang (*Curcuma longa* L.)	Rhizome	TCM Compounds	Occludin,TNF-α,LPS	[88]
	Fructus Aurantii (Quzhou origin)	Zhishi (*Citrus* × *aurantium* L.)	Immature fruit	TCM Ingredients	ZO-1,Occludin,M1 macrophages	[89]
	Panax Notoginseng Saponins	Sanqi (*Panax notoginseng* (Burk.) F.H.Chen)	Root and rhizome	TCM Ingredients	ZO-1,Claudin-1,SCFAs,TNF-α,IL-6	[90]
	Gypenosides	Jiaogulan (*Gynostemma pentaphyllum* (Thunb.) Makino)	Whole plant	TCM Ingredients	Occludin,ZO-1,LPS	[92]
	Volatile Oil of Amomum villosum	Sharen (*Amomum villosum* Lour.)	Mature fruit	TCM Ingredients	ZO-1,Occludin	[94]
	Salvianolic acid B	Danshen (*Salvia miltiorrhiza* Bunge)	Root and rhizome	TCM Compounds	Occludin,ZO-1	[95]
	Geniposide and Chlorogenic Acid Combination	Zhizi (*Gardenia jasminoides* J.Ellis) Jinyinhua (Lonicera japonica Thunb.) and Juhua (Chrysanthemum morifolium Ramat.)	Mature fruit	TCM Compounds	RhoA/ROCK,LPS,D-lactate	[60]
	Poria cocos polysaccharides	Fuling (*Poria cocos* (Schw.) Wolf)	Sclerotium	TCM Ingredients	PARP-1,Occludin,ZO-1	[98]
Regulate the level of bile acids	Hyperoside	Jinsitao (*Hypericum*)	Whole plant	TCM Compounds	FXR,LXR Aα,CYP7A1	[108]
	Nuciferine	Lianzi (*Nelumbo nucifera Gaertn.*(Lotus Plumule))	Embryo of mature seed (Lotus Plumule)	TCM Compounds	CYP7A1,CYP27A1,FXR	[110]
	Yellow tea polysaccharides	Huangcha (*Camellia sinensis* var. *huangcha*)	Young leaves	TCM Ingredients	CYP7A1,CYP27A1,FXR	[111]
	Ferulic Acid	Danggui (*Angelica sinensis* (Oliv.) Diels)	Root	TCM Compounds	PPARα,CYP7A1	[113]
	Quercetin	Hongdoushan (*Taxus spp*.)	Branches and leaves	TCM Compounds	mTOR/YY1,CYP7A1	[115]
	Nobiletin	Citrus fruits	Fruit	TCM Compounds	FXR,SHP,CYP7A1,CYP27A1	[116]
	Gypensapogenin A-Liposomes	Jiaogulan (*Gynostemma pentaphyllum* (Thunb.) Makino)	Whole plant	TCM Ingredients	FXR,CYP7A1,CYP8B1,CYP27A1	[117]
	Ganoderma Lucidum Polysaccharide Peptide	Lingzhi (Ganoderma lingzhi Sheng H. Wu et al.)	Fruiting body	TCM Ingredients	CYP7A1,CYP8B1,FXR-SHP/FGF	[118]
Regulate the gut microbiota	Chlorogenic Acid	Jinyinhua (*Lonicera japonica* Thunb)、Juhua (*Chrysanthemum morifolium* Ramat.)	Flower buds or flowers with initial opening	TCM Ingredients	Bifidobacterium,Escherichia coli,Occludin,ZO-1,LPS	[132]
	*Gynostemma pentaphyllum* (Thunb.) Makino	Jiaogulan (*Gynostemma pentaphyllum* (Thunb.) Makino)	Whole plant	TCM Ingredients	The ratio of Firmicutes to Bacteroidetes,Lactococcus spp,Ruminococcus spp	[133]
	*Gynostemma pentaphyllum* (Thunb.) Makino extract				Akkerrmansia,Klebsiella	[134]
	Gypenosides				The ratio of Firmicutes to Bacteroidetes	[135]
	Poria cocos polysaccharides	Fuling (*Poria cocos* (Schw.) Wolf)	Sclerotium	TCM Ingredients	Faecalibaculum,TLR4,NF-κB	[136]
	Seabuckthorn polysaccharides	Shaji (*Hippophae rhamnoides* L.)	Fruit	TCM Ingredients	Lentisphaerae, Firmicutes, Tenericutes,Peptococcus sp., RC9_gut_group sp., Parabacteroides sp.	[138]
	Atractylodes macrocephala extract BZEP self-microemulsion	Baizhu (*Atractylodes macrocephala* Koidz.)	Rhizome	TCM Ingredients	Lactobacillus, norank_f_Muribaculaceae,unclassified_f_Lachnospiraceae,Blautia	[139]
	Ophiopogon japonicus polysaccharides	Maidong (*Ophiopogon japonicus* (L. f.) Ker-Gawl)	Tuberous root	TCM Ingredients	Akkermansia muciniphila	[141]
	Tetrastigma hemsleyanum leaf extracts	Sanyeqing (*Tetrastigma hemsleyanum* Diels et Gilg)	Leaves	TCM Ingredients	Lactobacillale, Ruminococcaceae, Bifidobacteriales	[143]
	Tanshinone IIA	Danshen (*Salvia miltiorrhiza* Bunge)	Root and rhizome	TCM Compounds	Firmicutes, Actinobacteriotawere,Bacteroidota,Verrucomicrobiota	[145]
	Cassiae Semen	Juemingzi (*Cassia obtusifolia* L.)	Mature seed	TCM Ingredients	Firmicutes,Bacteroidetes,Proteobacteria	[147]
	Myricetin	onions, berries, grapes, and red wine	Fruit	TCM Compounds	butyric-acid-related gut microbiota,LPS/TLR4/NF-κB	[148]
	Polysaccharides Extracted from Old Stalks of *Asparagus officinalis* L	Lusun(*Asparagus officinalis* L.)	Stem	TCM Ingredients	butyric-acid-related gut microbiota,LPS/TLR4/NF-κB	[149]
Regulate immune function	Polydatin	*Huzhang (Polygonum cuspidatum* Sieb. et Zucc.)	Rhizome and root	TCM Compounds	CD68 macrophage,TLR4/NF-κB	[163]
	Quercetin	Hongdoushan (*Taxus spp*.)	Branches and leaves	TCM Compounds	TLR4/NF-κB	[164]
	Gynostemma pentaphyllum polysaccharides	Jiaogulan (*Gynostemma pentaphyllum* (Thunb.) Makino)	Whole plant	TCM Ingredients	TLR2/NLRP3,TNF-α,IL-1β	[167]
	A flavonoid-rich extract of bergamot juice	Foshou (*Citrus medica L. var. sarcodactylis* Swingle)	Fruit	TCM Ingredients	TLR4/MyD88/NF-κB/MAPK,TNF-α,IL-6	[168]
	Green tea polyphenol epigallocatechin-3-gallate	Chashu (*Camellia sinensis* (L.) O. Ktze.)	Young leaves	TCM Compounds	IgA^+^B cells	[170]

**Table 2:** Traditional Chinese medicine formulas affects NAFLD by regulating the intestinal barrier

Mechanism	TCM	Target / medium	Reference
Restore the intestinal mechanical barrier and mitigate chronic inflammatory response	Ginkgo biloba preparations	ZO-1, Occludin,Claudin-1,TNF-α,LPS	[100]
	Zuogui Jiangtang Qinggan Prescription	ZO-1,Occludin,Claudin-1	[102]
	Chaihu Guizhi Ganjiang Decoction	ZO-1,Occludin,Claudin-1,PPARα	[104]
	Huazhi Rougan Granule	ZO-1,Occludin,LPS	[106]
Regulate the level of bile acids	Jiangzhi Granule	FXR,VDR	[123]
	Hua Zhi Rou Gan Granules	OSTβ,LCA,HCA,βDCA	[124]
	Qige Decoction	SIRT6-PPARα	[126]
	Zhuyu Pill	FXR,CYP7A1	[129]
	Ling-Gui-Zhu-Gan Decoction	SCFA,PPARα	[131]
Regulate the gut microbiota	Zhishi Daozhi Decoction	The ratio of Firmicutes to Bacteroidetes,SCFA	[152]
	Xiezhuo Tiaozhi Formula	Ileibacterium valens	[154]
	The spleen-strengthening and liver-draining herbal Formula	Desulfobacterota,Firmicutes,Bacteroidetes	[155]
	Si Miao Formula	Akkermansia muciniphila	[157]
	Erchen Decoction	Lactobacillus, Bifidobacterium,Butyricicoccus,Bacteroides,Parabacteroides,Sediminibacterium,SCFA	[159]
	Shenling Baizhu Powder Herbal Formula	SCFA,Bifidobacterium,Anaerostipes	[162]
Regulate immune function	Dahuang Zhechong Pill	The ratio of Th17/Treg,IL-17,hs-CRP,TNF-α	[173]
	Fuzi-Lizhong Decoction	TLR4/MyD88/TRAF6,IL-2,IL-6,TNF-α	[176]
	Qianggan extract	immune-related mRNAs (e.g., Cd28, Cd8a, Il15, and Klrk1)	[178]
	A decoction of Chinese thorowax root, Scutellaria root, and White peony root combination	TNF-α,TGF-β,NF-κB,TLR4,SIgA	[180]

Pharmacological studies collectively demonstrate that TCM ingredients and compounds ameliorate intestinal barrier dysfunction in NAFLD through conserved mechanisms. Curcumin is a compound extracted from the Jianghuang (Curcuma longa L.). Study demonstrated that curcumin restores tight junction/desmosome ultrastructure and attenuates organelle damage in HFD-induced rats with NAFLD, concomitant with elevated Occludin expression and reduced serum TNF-α/LPS [88]. In a Reg3g-deficient murine NASH model, Zhishi (*Citrus* × *aurantium* L., Quzhou origin) was found to upregulate ZO-1 and Occludin, which may contribute to counteracting increased intestinal permeability. This was associated with reduced endotoxin translocation and subsequent suppression of M1 macrophage polarization [89]. This suggests that enhancing intestinal barrier integrity is a potential mechanism through which Zhishi may alleviate NASH. Panax notoginseng saponins, bioactive ingredients derived from the roots of Sanqi (*Panax notoginseng* (Burk.) F.H.Chen), represent the principal triterpenoid glycosides responsible for its pharmacological activities. In HFD-induced obese mouse models, Panax notoginseng saponins were shown to ameliorate hepatic steatosis and fibrosis. This hepatoprotective effect is associated with the modulation of the gut-liver axis in a TLR4-dependent manner, including the restoration of intestinal barrier proteins (Claudin-1, ZO-1). Crucially, the protective effects of Panax notoginseng saponins were abolished by the TLR4 agonist LPS, providing key evidence for the central role of TLR4 signaling in this pathway [90]. Similarly, Gypenosides, the principal ingredients extracted from Jiaogulan (*Gynostemma pentaphyllum* (Thunb.) Makino), represent a class of dammarane-type triterpenoid saponins with demonstrated metabolic regulatory activities [91]. Cell and animal studies indicate that gypenosides can effectively attenuate hepatic steatosis associated with MAFLD by concomitantly activating AMPK signaling and inhibiting the TLR4/NF-κB inflammatory pathway. The mechanism involves protection of the intestinal barrier, including the reversal of LPS-induced decreases in tight junction proteins (Occludin, ZO-1) and increased permeability [92]. The volatile oil of A. villosum refers to the complex mixture of volatile constituents extracted from Sharen (*Amomum villosum* Lour.), which demonstrates pharmacological activities including facilitation of gastrointestinal digestion, anti-inflammatory effects, and antimicrobial properties [93]. LU et al. discovered that the volatile oil of A. villosum promoted the expression of ZO-1 and Occludin proteins and inhibited the TLR4/NF-κB signaling pathway. These coordinated changes collectively suggest its potential to ameliorate chronic low-grade inflammation in the liver, contributing to the observed preventive and therapeutic effects against NAFLD in their model [94]. A study in HFD-induced NASH rats demonstrated that Salvianolic acid B from Danshen (*Salvia miltiorrhiza* Bge.) extract treatment significantly alleviated liver injury (reduced ALT, AST, TG, TC and NAS score) and systemic endotoxemia (lowered ET, DAO). These improvements were associated with the restoration of intestinal barrier integrity, evidenced by recovered expression of Occludin and ZO-1 and normalized ultrastructure of intestinal junctions. The study concluded that Sal B exerts hepatoprotection by restoring healthy intestinal barrier function [95]. Geniposide, an iridoid glycoside primarily derived from Zhizi (*Gardenia jasminoides* J.Ellis), demonstrates significant anti-inflammatory and hepatoprotective effects while modulating metabolic homeostasis. Chlorogenic acid, a phenolic acid compound extracted from traditional Chinese medicinal herbs including Jinyinhua (*Lonicera japonica* Thunb.) and Juhua (*Chrysanthemum morifolium* Ramat.), demonstrates significant anti-inflammatory, antihyperglycemic, and antihyperlipidemic pharmacological activities [96, 97]. The combination of geniposide and chlorogenic acid reduced gut-derived LPS signaling, decreased plasma levels of LPS and D-lactic acid, restored colonic tight junction expression by downregulating RhoA/ROCK signaling, exerted intestinal barrier protective functions, and alleviated hepatic steatosis and inflammation in HFD-induced NASH model mice [60]. Poria cocos polysaccharides, a class of heteropolysaccharides extracted from the sclerotium of Fuling (*Poria cocos* (Schw.) Wolf), represent one of the principal ingredients responsible for its pharmacological activities. Studies have shown that pyroptosis of intestinal macrophages can occur in HFD-induced NAFLD, and Poria cocos polysaccharides effectively reduced pyroptosis-driven intestinal vascular barrier disruption by regulating PARP-1, increased the expression of Occludin and ZO-1, reduced endotoxin translocation, and alleviated the progression of NAFLD [98].

Ginkgo biloba preparations (GBP), was proposed by Professor Pan Shuhua from Nanjing University of Chinese Medicine. This preparation is composed of the extracts of loquat and ginkgo leaves. It can effectively restores intestinal tight junctions and ameliorates intestinal barrier dysfunction. This action reduces endotoxin translocation, subsequently attenuating inflammatory responses in hepatocytes and ultimately repairing liver injury [99]. Researches show that GBP significantly upregulated ZO-1 and occludin expression, concurrently reducing serum TNF-α, LPS, AST, TG, and CHOL levels while alleviating hepatic steatosis [100].

Zuogui Jiangtang Qinggan Prescription (ZGJTQGP), innovatively developed by researchers at Hunan University of Chinese Medicine based on the classical prescription Zuogui Pill from Zhang Jingyue's Jingyue Complete Works (Ming Dynasty, 1368–1644), is a modern compound specifically designed for treating type 2 diabetes mellitus (T2DM) comorbid with NAFLD. This prescription comprises 10 medicinal components: Shudihuang (*Rehmanniae Radix Praeparata*), Huangqi (*Astragali* Radix), Shanyao (*Dioscorea opposita* Thunb), Shanzhuyu (*Cornus officinalis* Siebold & Zucc), Huanglian (*Coptis chinensis* Franch), Danshen (*Salvia miltiorrhiza* Bunge), Yinchen (*Artemisia scoparia* Waldst. et Kit), Huzhang (*Polygonum cuspidatum* Sieb. et Zucc), Wenyujin (*Curcuma wenyujin* Y. H. Chen et C. Ling), and Chenpi (*Citrus reticulata* Blanco). ZGJTQGP effectively ameliorates glucolipid metabolic disorders [101]. Mechanistic studies elucidate that it enhanced intestinal mucosal repair through triple upregulation of tight junction proteins (ZO-1/occludin/claudin-1), increased villus height and goblet cell density, and maintained microbiota homeostasis [102].

Chaihu Guizhi Ganjiang Decoction (CHGZGJD), originating from Article 147 of the classical Chinese medical text Shanghan Lun (Treatise on Cold Damage Disorders), comprises seven medicinal components: Beichaihu (*Bupleurum chinense* DC), Huangqin (*Scutellaria baicalensis* Georgi), Rougui (*Cinnamomum cassia* Presl), Shengjiang (*Zingiber officinale* Rosc), Gualou (*Trichosanthes kirilowii* Maxim), Muli (*Ostrea gigas* Thunb), and Gancao (*Glycyrrhiza uralensis* Fisch). Modern clinical studies confirm its multi-target efficacy in improving hepatic function, exerting antioxidative, anti-inflammatory, and anti-fibrotic effects [103]. Mechanistic investigations demonstrate that CHGZGJD decreased intestinal permeability and plasma inflammatory mediators (LPS, TNF-α, IL-6, IL-1β, MCP-1) via tight junction protein enhancement, subsequently attenuating hepatic inflammation and steatosis through PPARα-mediated fatty acid oxidation [104].

Huazhi Rougan Granule (HZRGG) was approved in China (2009) for treating non-alcoholic simple fatty liver with Shi-Re Zhong-Zu syndrome (dampness-heat obstructing middle energizer). Its primary functions include clearing heat, draining dampness, resolving turbidity, detoxifying, removing blood stasis, and softening the liver. The formulation contains 16 medicinal components: Yichen (*Artemisia scoparia* Waldst. et Kit), Juemingzi (*Cassia obtusifolia* L), Dahuang (*Rheum palmatum* L), Zexie (*Alisma plantago-aquatica* L. var. *orientale* Samuels), Zhuling (*Polyporus umbellatus* (Pers.) Fries), Cangzhu (*Atractylodes lancea* (Thunb.) DC), Baizhu (*Atractylodes macrocephala* Koidz), Chenpi (*Citrus reticulata* Blanco), Gualou (*Trichosanthes kirilowii* Maxim), Nvzhenzi (*Ligustrum lucidum* Ait), Hanliancao (*Eclipta prostrata* L), Gouqizi (*Lycium barbarum* L), Xiaoji (*Cirsium setosum* (Willd.) MB), Beichaihu (*Bupleurum chinense* DC), and Gancao (*Glycyrrhiza uralensis* Fisch).[105]. Research demonstrates that this granule suppressed the miR-122/TLR4/MyD88/NF-κB axis to elevate ZO-1/occludin expression, reduce serum LPS, and ameliorate intestinal injury and hepatic inflammation [106].

The core mechanism of this section centers on TCM-mediated repair of the intestinal mechanical barrier and inhibition of chronic inflammation. Discrepancies across studies arise from variations in TCM extraction methods, experimental model durations, and pathway dependency. Nevertheless, "enhancing epithelial integrity and suppressing inflammatory cascades" represents a consensus core pathway, providing a clear molecular basis for TCM-targeted therapy of NAFLD via the intestinal mechanical barrier.

### Modulation of bile acid homeostasis

The pathogenesis of NAFLD is closely associated with dysregulated lipid metabolism and disrupted bile acid homeostasis. Accumulating evidence indicates that both bioactive ingredients/compounds and compound formulas of TCM exert therapeutic effects through the multilevel regulation of the bile acid metabolic axis (Tables 1 and 2), thereby establishing a novel multitarget therapeutic framework for NAFLD intervention. Specifically, TCM-derived bioactive components, such as flavonoids, polysaccharides, and alkaloids, can directly target key regulators of bile acid metabolism—including FXR, PXR, and CYP7A1—modulating bile acid synthesis, transport, and enterohepatic cycling to restore their physiological profiles. This restoration of bile acid homeostasis alleviates intrahepatic cholestasis, reduces hepatic lipid accumulation, and suppresses pro-inflammatory responses. Such a multicomponent-multitarget mode of action exemplifies TCM’s unique advantage in reprogramming metabolic pathways via bile acid-centric network pharmacology, highlighting its potential as a promising therapeutic strategy for NAFLD.

Hyperoside is a ubiquitously distributed flavonol glycoside in medicinal plants, demonstrating potent anti-inflammatory and antioxidant activities [107]. WANG et al. found that hyperosid elevates hepatic FXR/LXRα expression and CYP7A1 activity in rats, shifting bile acid composition toward conjugated forms to ameliorate NAFLD [108]. Nuciferine, a bisbenzylisoquinoline alkaloid extracted from the embryo of Lianzi (*Nelumbo nucifera* Gaertn.(Lotus Plumule)), demonstrates multimodal pharmacological activities including anti-fibrotic, anti-inflammatory, and metabolic regulatory effects [109]. Yellow Tea Polysaccharides are water-soluble heteropolysaccharides extracted from the leaves of Huangcha (*Camellia sinensis var. Huangcha)*. Researches show that nuciferine and yellow tea polysaccharides promote CYP7A1/CYP27A1-mediated bile acid synthesis while inhibiting ileal FXR signaling, thereby enhancing fecal bile acid excretion and improving hepatic lipid metabolism in HFD-induced NAFLD [110, 111]. Ferulic acid is a ubiquitous phenolic phytochemical belonging to the hydroxycinnamic acid class, primarily extracted from Danggui (*Angelica sinensis* (Oliv.) Diels), Chuanxiong (*Ligusticum* chuanxiong Hort), and cereal brans (rice, wheat) [112]. Research has found that ferulic acid upregulates PPARα and modulates CYP7A1, conferring protection against iron overload-induced metabolic dysregulation in vivo and in vitro [113]. Quercetin is a type of polyphenolic flavonoid found in fruits and vegetables. It has the effects of clearing heat and detoxifying, cooling blood and stopping bleeding. Studies have confirmed that NAFLD can be treated through anti-inflammatory, anti-oxidative stress and lipid metabolism regulation [114]. In T2DM-associated NAFLD, quercetin activates CYP7A1 transcription via mTOR/YY1 pathway suppression, restoring cholesterol homeostasis through enhanced bile acid conversion [115]. Nobiletin is a flavonoid compound abundant in citrus fruits with drying dampness and eliminating phlegm effects. It has been proven to be able to treat NAFLD by exerting anti-inflammatory, antioxidant and regulating fat metabolism effects. Xu et al. found that nobiletin ameliorates MAFLD by downregulating hepatic FXR/SHP signaling while upregulating CYP7A1/CYP27A1 expression, thereby accelerating hepatic cholesterol conversion [116]. Gypensapogenin A-liposomes is a drug delivery system formed by encapsulating the gypenoside A in liposomes. Researches show that gypensapogenin A-liposomes activate FXR to inhibit CYP7A1/CYP8B1 yet induce CYP27A1, reducing total bile acid pool while increasing chenodeoxycholic acid content, ultimately lowering serum total cholesterol (TC) / triglycerides (TG) levels in NAFLD [117]. Ganoderma lucidum polysaccharide peptide is an active ingredient extracted from Lingzhi (Ganoderma lingzhi Sheng H. Wu et al.). It elevates CYP7A1/CYP8B1 expression in murine NAFLD models, activating FXR-SHP/FGF-dependent pathways to suppress fatty acid synthesis, which reduces lipid droplet accumulation and triglyceride content in both OA/PA-induced HepG2 cells and primary hepatocytes [118].

Jiangzhi Granule (JZG), designed by Shanghai University of Traditional Chinese Medicine based on the etiology and pathogenesis of NAFLD, exerts therapeutic effects by clearing heat and draining dampness. Composed of Jiaogulan (*Gynostemma pentaphyllum* (Thunb.) Makino), Huzhang (*Polygonum cuspidatum Sieb. et Zucc*), Danshen (*Salvia miltiorrhiza* Bunge), Yinchen (*Artemisia scoparia* Waldst. et Kit), and Gancao (*Glycyrrhiza uralensis* Fisch), its efficacy and safety in treating NAFLD have been empirically validated in both clinical studies and in vivo/in vitro experimental research [119–122]. Mechanistically, JZG modulates the bile acid profile and activates the intestinal FXR. It upregulates intestinal VDR levels in NASH mouse models, increases the proportion of secondary bile acids, and restores intestinal barrier integrity [123].

Hua Zhi Rou Gan Granules (HZRGG) can reduce hepatic steatosis and inflammation in NASH mouse induced by a methionine-choline deficient (MCD) diet, decrease hepatic AST and ALT levels, and their mechanism may be related to enhancing fecal bile acid excretion, reducing the reabsorption of secondary bile acids, and inhibiting the expression of ileal bile acid transporters and organic solute transporter β subunit [124].

Qige Decoction (QG) is an empirical formula developed by Professor Keer Huang, a renowned Traditional Chinese Medicine (TCM) practitioner in Guangdong Province. It is composed of Huangqi (*Astragalus mongholicus* Bunge), Gegen (*Pueraria montana* var. *lobata* (Willd.) Maesen & S.M.Almeida ex Sanjappa & Predeep), and Zhishi (*Citrus* × *aurantium* L) in a 6:2:1 ratio. Its primary therapeutic actions are to fortify the spleen, replenish qi, dry dampness, and resolve turbidity. Clinically, QG is primarily indicated for NAFLD presenting with the TCM pattern of spleen deficiency with dampness encumbrance [125]. Sirtuin 6 (SIRT6) interacts with PPARα to regulate fatty acid oxidation. Downregulation of SIRT6 can inhibit PPARα-mediated fatty acid oxidation and exacerbate lipid accumulation in hepatocytes. Studies have found that Qige Decoction can upregulate the SIRT6-PPARα signaling pathway in both in vivo and in vitro experiments, promote the expression of genes related to fatty acid oxidation, alleviate dyslipidemia, and reduce hepatic steatosis [126].

Zhuyu Pill (ZYP, also known as Zuojin Wan), originating from the Taiping Shenghui Fang (Imperial Grace Formulary) compiled by Wang Huaiyin, Wang You, and others during the Song Dynasty, is composed of Huanglian (*Coptis chinensis* Franch) and Wuzhuyu (*Tetradium ruticarpum* (A. Juss.) T.G. Hartley). Its primary therapeutic effects are to invigorate spleen yang and soothe the liver to regulate qi. It is clinically used to treat various metabolism-related diseases, including NAFLD, atherosclerosis, and hyperlipidemia [127, 128]. Recent studies demonstrate that ZYP effectively ameliorates serum lipid levels, reduces inflammatory responses, and attenuates hepatocyte injury and lipid accumulation in mice with HFD-induced NAFLD. The underlying mechanism involves inhibiting signaling transduction via the ileal FXR and Fibroblast Growth Factor 15 (FGF15), subsequently enhancing the expression of CYP7A1. This action promotes BA synthesis and increases the metabolic elimination of cholesterol, thereby modulating BA metabolism [129].

Ling-Gui-Zhu-Gan Decoction (LGZGD), first documented in Zhang Zhongjing's Shang Han Za Bing Lun (Treatise on Cold Damage and Miscellaneous Diseases), is composed of four medicinal herbs: Fuling (*Poria cocos* (Schw.) Wolf), Rougui (*Cinnamomum cassia* (L.) J.Presl), Baizhu (*Atractylodes macrocephala* Koidz), and Gancao (*Glycyrrhiza uralensis* Fisch). Its primary therapeutic effects are to warm yang to resolve fluid retention and fortify the spleen to promote water disinhibition. Clinically, LGZGD has demonstrated significant efficacy in the treatment of NAFLD [130]. Recent studies reveal that Linggui Zhugan Decoction significantly ameliorates hepatic dysfunction and lipid accumulation in mice with HFD-induced NAFLD. The underlying mechanism involves altering the BA profile, increasing the concentration of SCFAs, and modulating the expression of genes in the liver associated with both primary bile acid biosynthesis pathways and the PPAR signaling pathway [131].

TCM modulates bile acid homeostasis through multiple targets to ameliorate NAFLD, with core targets including CYP7A1, FXR, and bile acid transport-related molecules. TCM can regulate bile acid homeostasis through the entire "synthesis-transport-signaling" cascade, and its multi-target characteristic is compatible with the complexity of bile acid metabolism.

### Regulation of gut microbiota

Bioactive ingredients/compounds and compound formulas of TCM can drive the compositional restructuring of gut microbial communities and regulate the gut microbiota metabolic network through multiple pathways. On one hand, they restore microbial diversity, enrich beneficial probiotic taxa (e.g., Bifidobacterium spp., Akkermansia spp.) to rectify gut dysbiosis, and simultaneously promote the production of SCFAs. This cascade of effects synchronously enhances intestinal barrier function, suppresses endotoxin translocation, and alleviates hepatic inflammatory responses as well as lipid metabolism disorders. Furthermore, they further block the progression of NAFLD through the synergistic regulation of the enterohepatic bile acid axis. This process acts in synergy with the gut microbiota regulatory mechanism to collectively improve the therapeutic efficacy (Tables 1 and 2).

Chlorogenic acid not only exerts the efficacy of improving NAFLD by restoring the intestinal mechanical barrier. A study investigated the multifaceted protective effects of chlorogenic acid on HFD-induced NAFLD in mice. The results demonstrated that chlorogenic acid alleviated hepatic steatosis and inflammation and inhibited the hepatic TLR4 inflammatory pathway. These effects were accompanied by changes at the intestinal level: an increase in fecal Bifidobacterium, a decrease in Escherichia coli, and an upregulation of tight junction proteins (Occludin and ZO-1). Concurrently, a decrease in LPS levels and an increase in GLP-1 levels were observed in the portal vein. Synthesizing these coordinated changes, the authors propose that the hepatoprotective effect of chlorogenic acid may be associated with its anti-inflammatory effects, which could be linked to the modulation of gut microbiota, enhancement of intestinal barrier function, and increased GLP-1 secretion [132]. In HFD-induced NAFLD rats, the hepatoprotective effect of Jiaogulan (Gynostemma pentaphyllum (Thunb.) Makino) has been correlated with specific modulations of the gut microbiota (e.g., a reduced Firmicutes/Bacteroidetes ratio, increased Lactococcus, decreased Ruminococcus) and the preservation of intestinal barrier integrity, pointing to microbiota-mediated pathways as a likely component of its action [133]. Furthermore, in NASH mice, treatment with Jiaogulan (Gynostemma pentaphyllum (Thunb.) Makino) extract was accompanied by a regulated gut microbiota composition, including an increase in beneficial bacteria such as Akkermansia and a decrease in harmful bacteria such as Klebsiella. This shift co-occurred with reduced liver inflammation, hepatocyte lipid accumulation, and lipid peroxidation [134]. Separately, gypenosides, active ingredients of Jiaogulan, were shown to lower the fecal Firmicutes/Bacteroidetes ratio in NAFLD mice, an effect that was associated with the mitigation of fatty liver hepatitis [135]. These parallel observations suggest a potential link between Jiaogulan-induced microbiota remodeling and its hepatoprotective outcomes. A systematic study demonstrated that Poria cocos polysaccharides effectively prevented methionine- and choline-deficient diet-induced NASH in mice, significantly alleviating liver injury, dysfunction, and oxidative stress. This protective effect was accompanied by a remodeling of the gut microbiota—such as increasing the abundance of Faecalibaculum and reducing endotoxin load—and the downregulation of multiple hepatic inflammatory and immune-related pathways, including the TLR4/NF-κB/CCL3/CCR1 axis. Together, these results suggest that the effect of Poria cocos polysaccharides may be related to its modulation of the gut microbiota and subsequent suppression of downstream inflammatory signaling [136]. Seabuckthorn polysaccharides are a class of bioactive polysaccharides extracted from Shaji (*Hippophae rhamnoides* L.), which possess the efficacy of eliminating phlegm to relieve cough, promoting digestion to resolve food stagnation, and activating blood circulation to dissipate blood stasis. In a mouse model of HFD-induced obesity, seabuckthorn polysaccharides was shown to ameliorate metabolic parameters and hepatic steatosis. This effect co-occurred with a reorganization of the gut microbiota—such as increasing beneficial Bifidobacterium and Bacteroides—and an increase in fecal SCFAs, suggesting that it's action may involve the regulation of the gut microbiome and SCFA production along the gut-liver axis [137]. A study demonstrated that seabuckthorn polysaccharide was more effective than probiotic control in ameliorating hepatic lipid accumulation in HFD-induced NAFLD mice, and its effect was closely associated with the remodeling of the gut microbiota. The intervention, while alleviating hepatic steatosis and inflammation, was accompanied by significant changes in the abundance of specific intestinal bacteria such as Lentisphaerae and Peptococcus, which were correlated with hepatic oxidative stress markers [138]. The Atractylodes macrocephala extract BZEP self-microemulsion is a novel drug delivery system prepared by self-microemulsifying technology, with the extract of the TCM Baizhu (*Atractylodes macrocephala* Koidz.) as its active ingredient. It possesses the effects of invigorating the spleen and replenishing qi, as well as drying dampness and promoting diuresis. LI et al. found that the atractylodes macrocephala extract BZEP self-microemulsion alleviated MAFLD in rats via the gut-liver axis [139]. This intervention increased beneficial bacteria (e.g., Lactobacillus) while suppressing harmful bacteria, upregulated intestinal tight junction proteins (Occludin and Claudin-1) and serum high-density lipoprotein cholesterol (HDL-C) levels, and reduced LPS content alongside TLR4/MyD88/NF-κB pathway activation, thereby restoring hepatic histopathology. Similarly, Ophiopogon japonicus polysaccharides (OJP), a class of water-soluble polysaccharide ingredients, are extracted from Maidong (*Ophiopogon japonicus* (L. f.) Ker-Gawl.). They possess the effects of nourishing yin and promoting the production of body fluid, as well as moistening the lung and clearing the heart. It has been confirmed that they can regulate the intestinal microbiota [140]. Studies have found that Ophiopogon japonicus polysaccharides inhibited NAFLD progression in HFD-fed mice by altering gut microbiota structure/diversity, enhancing Akkermansia muciniphila abundance, and regulating lipid-metabolic pathways, ultimately improving hepatic steatosis, lipid accumulation, and chronic inflammation [141]. "Tetrastigma hemsleyanum leaf extracts" refers to the leaf extract of the plant known as " Sanyeqing (*Tetrastigma hemsleyanum* Diels et Gilg) ". Tetrastigma hemsleyanum leaf extracts possess the TCM effects of clearing heat and detoxifying, as well as resolving phlegm and dissipating masses. Studies have demonstrated their potential efficacy in alleviating hyperlipidemia and preventing oxidative stress [142]. Tetrastigma hemsleyanum leaf extracts elevated the relative abundance of Lactobacillus, Ruminococcaceae, and Bifidobacterium in NAFLD mice, preserved intestinal barrier integrity, and mitigated NAFLD-associated oxidative stress, lipid accumulation, and inflammation [143]. Tanshinone IIA is derived from Danshen (*Salvia miltiorrhiza* Bunge), as one of the primary compounds in this herb. In the context of TCM, it possesses the effects of promoting blood circulation to remove blood stasis, cooling blood, detoxifying, and resolving abscesses. Studies show that tanshinone IIA can effectively regulate the intestinal microbiota and improve lipid metabolism [144]. Recent research found that tanshinone IIA ameliorated hepatic steatosis, normalized blood lipid profiles, and improved glucose tolerance in NAFLD mice by restoring gut microbiota dysbiosis—specifically reducing Firmicutes and Actinobacteria while significantly increasing Bacteroidetes and Verrucomicrobia [145]. Cassiae semen extract is derived from the dried ripe seeds of Juemingzi (*Cassia obtusifolia* L.). In TCM, it exhibits the effects of clearing liver heat to improve eyesight and moistening the intestines to promote defecation. Studies have revealed that Cassiae semen extract can regulate intestinal microbiota, lipid metabolism, and immune function, thereby ameliorating hyperlipidemia in rats [146]. Notably, LUO et al. demonstrated that Cassiae semen extract attenuated lipid accumulation, intestinal barrier disruption, hepatic injury, and inflammation in HFD-induced NAFLD mice through gut microbiota modulation. Critically, antibiotic-induced microbiota ablation abolished these therapeutic effects, indicating that the efficacy of Cassiae semen extract against NAFLD is microbiota-dependent [147]. Sun et al. demonstrated that supplementation with myricetin and polysaccharides extracted from old stalks of Lusun(*Asparagus officinalis* L.) could reduce hepatic lipid synthesis and inflammation, as well as retard the development NAFLD in high-fat diet-fed rats. This therapeutic effect was associated with the modulation of butyrate-related gut microbiota in feces, enhancement of intestinal barrier function, and inhibition of the LPS/TLR4/NF-κB pathway. Furthermore, this effect was verified in fecal microbiota transplantation experiments [148, 149]. The modulation of the gut microbiota by active ingredients of TCM and natural compounds to ameliorate NAFLD has become a significant research direction. However, most current studies remain at the level of correlative observations—that is, the concurrent observation of microbial changes and liver disease improvement—without rigorously demonstrating that alterations in the microbiota are the necessary cause for the therapeutic effects of these herbal components.

Zhishi Daozhi Decoction (ZSZD), originating from Nei Shang Wai Gan Bian (Differentiation on Endogenous and Exogenous Diseases) authored by Dong-yuan Li in 1247, is composed of eight distinct Chinese medicinal materials: Zhishi (*Citrus* × *aurantium* L), Dahuang (*Rheum palmatum* L), Huanglian (*Coptis chinensis* Franch), Huangqin (*Scutellaria baicalensis* Georgi), Shenqu (Massa Medicata Fermentata), Baizhu (*Atractylodes macrocephala* Koidz), Fuling (*Poria cocos* (Schw.) Wolf), and Zexie (*Alisma orientale* (Sam.) Juz). ZSZD is commonly employed clinically for the treatment of NAFLD and is recognized to exert modulatory effects on the gut microbiota [150, 151]. Research by Bi et al. demonstrated that in mice with HFD-induced NAFLD, ZSZD significantly reduced serum levels of TG, CHO, ALT, and AST, and attenuated hepatic lipid accumulation. The underlying mechanism is associated with the modulation of gut microbiota composition, increased intestinal levels of SCFAs, and restoration of intestinal barrier function [152].

Xiezhuo Tiaozhi Formula (XZTZ), a modified formulation derived from Zexie Decoction in the "Synopsis of the Golden Chamber—Phlegm and Cough Disease", is typically composed of several Chinese medicinal herbs: Zexie (*Alisma plantago-aquatica* var. orientale Sam), stir-fried Baizhu (*Atractylodes macrocephala* Koidz), Fuling (*Poria cocos* (Schw.) Wolf), stir-fried Daidaihua (*Citrus aurantium* f. *daidai*), raw Shanzha (*Crataegus pinnatifida* var. major N.E.Br), and Lianzi (*Nelumbo nucifera* Gaertn). XZTZ inhibits lipid accumulation and reduces inflammatory injury in hepatocytes [153]. Research has revealed that its mechanism of action involves upregulating the abundance of the gut bacterium Ileibacterium valens. This leads to increased levels of the purine nucleoside inosine, which significantly enhances fatty acid β-oxidation. Consequently, this cascade reduces the expression of pro-inflammatory cytokines and inhibits hepatocyte pyroptosis, thereby exerting a protective effect on the liver in NAFLD mouse models [154].

The Spleen-Strengthening and Liver-Draining Formula (SLF), developed based on the Traditional Chinese Medicine (TCM) theory of "One Qi Circulation" originating in the Qing Dynasty (1749), consists of Beichaihu (*Bupleurum chinense* DC), Baishao (*Paeonia lactiflora* Pall), Beishashen (*Glehnia littoralis* F.Schmidt ex Miq), Baizhu (*Atractylodes macrocephala* Koidz), Fuling (*Poria cocos* (Schw.) Wolf), Chenpi (*Citrus reticulata* Blanco), Gancao (*Glycyrrhiza uralensis* Fisch. ex DC), Chuipencao (*Sedum sarmentosum* Bunge), Shanzhatan (*carbonized Crataegus pinnatifida* Bge. var. major N.E.Br), and Danshen (*Salvia miltiorrhiza* Bunge). In a randomized controlled clinical trial, Hui et al. observed significantly elevated levels of the Firmicutes phylum and significantly reduced levels of the Bacteroidetes phylum in patients with abnormal ALT and AST. Their findings demonstrated that SLF increases gut microbial diversity and species richness in NAFLD patients [155].

The Si Miao Formula (SMF), a classical TCM formulation, originates from Cheng Fang Bian Du (Convenient Reading of Established Formulas) authored by Zhang Bingcheng during the Qing Dynasty. It comprises four medicinal herbs: Huangbai (P*hellodendron chinense* C.K.Schneid), Cangzhu (*Atractylodes lancea* (Thunb.) DC), Yiyiren (*Coix lacryma-jobi* L. var. ma-yuen (Rom.Caill.) Stapf), and Niuxi (*Achyranthes bidentata* Blume). SMF effectively reduces serum lipid levels and alleviates NAFLD [156]. Studies have demonstrated that in NAFLD mice induced by a high-fat/high-sugar diet, SMF can reduce body weight gain, alleviate hepatic steatosis, and decrease hepatic lipid content. Concurrently, SMF significantly alters the composition of gut microbiota, particularly increasing the proportion of Akkermansia muciniphila [157].

Erchen Decoction (ECD), originating from the Taiping Huimin Heji Jufang (Formulary of the Peaceful Benevolent Dispensary) compiled by Chen Shiwen during the Song Dynasty, is composed of Juhong (*Citrus reticulata* 'Chachi' Blanco), Banxia (*Pinellia ternata* (Thunb.) Breitenb), Fuling (*Poria cocos* (Schw.) Wolf), Gancao (*Glycyrrhiza uralensis* Fisch. ex DC), Wumei (*Prunus mume* (Siebold) Siebold & Zucc), and Shengjiang (*Zingiber officinale* Roscoe). ECD has been extensively utilized in the clinical treatment of NAFLD [158, 159]. Research demonstrates that Er Chen Decoction significantly increases intestinal levels of Lactobacillus, Bifidobacterium, and Butyricicoccus species, while downregulating the abundance of Bacteroides, Parabacteroides, and Parabacteroides distasonis in NAFLD rat models. Concurrently, ECD elevates total SCFAs and butyrate levels in both serum and liver tissue. These changes enhance hepatic fatty acid β-oxidation, thereby ameliorating obesity-associated hepatic steatosis and dyslipidemia [159].

Shenling Baizhu Powder Herbal Formula (SLP), a classical formula documented in the Song Dynasty's Taiping Huimin Hejiju Fang (Formulary of the Peaceful Benevolent Dispensary), is composed of ten medicinal components: Renshen (*Panax ginseng* C.A.Mey), Fuling (*Poria cocos* (Schw.) Wolf), Baizhu (*Atractylodes macrocephala* Koidz), Shanyao (*Dioscorea oppositifolia* L), Biandou (*Lablab purpureus* (L.) Sweet subsp. Purpureus), Lianzi (*Nelumbo nucifera* Gaertn), Gancao (*Glycyrrhiza uralensis* Fisch. ex DC), Yiyiren (*Coix lacryma-jobi* L. var. ma-yuen (Rom.Caill.) Stapf), Jiegeng (*Platycodon grandiflorus* (Jacq.) A.DC), and Sharen (*Amomum villosum* Lour. var. *xanthioides* (Wall. ex Baker) T.L.Wu & S.J.Chen). SLP effectively attenuates hepatic steatosis [160, 161]. Zhang et al. demonstrated that in a rat model of NAFLD induced by a high-fat diet, Shenling Baizhu San could increase the relative abundance of SCFAs-producing anaerobic bacteria such as Bifidobacterium spp. It significantly modulates the gut microbiota of NAFLD rats, reduces the level of LPS in the portal vein, and ultimately ameliorates hepatic inflammation and steatosis. [162].

This section clarifies that TCM exerts anti-NAFLD effects by reshaping gut microbiota structure and regulating microbial metabolites. TCM generally enriches beneficial bacteria such as Bifidobacterium, Lactobacillus, and Akkermansia, while inhibiting pro-inflammatory bacteria including Escherichia coli and Klebsiella to correct gut dysbiosis. Concurrently, it promotes the production of SCFAs, which provide energy for intestinal epithelial cells and strengthen tight junction function. However, most current studies remain at the level of correlational observations, and further validation is required to confirm the causal relationship between microbiota alterations and NAFLD amelioration. Overall, TCM-mediated gut microbiota regulation is characterized by "overall reshaping and key bacterial targeting", serving as one of the core mechanisms underlying TCM's treatment of NAFLD via the gut-liver axis.

### Regulation of immune function

TCM exerts prominent regulatory and ameliorative effects on intestinal immune function in NAFLD. Specifically, TCM ingredients/compounds and compound formulas modulate the activity of intestinal immune cells, stimulate the production of immunoglobulins (with a particular emphasis on sIgA), and regulate macrophage polarization. These coordinated actions collectively enhance intestinal mucosal barrier function, suppress the release of pro-inflammatory cytokines, and attenuate immune-mediated hepatic damage, thereby mitigating the pathogenesis of NAFLD. In recent years, accumulating attention has been directed toward the role of abnormal intestinal immune barrier function in driving NAFLD progression. Notably, TCM formulas, leveraging their inherent advantages of multi-component and multi-target actions, have demonstrated unique potential in regulating the intestinal immune barrier, further supporting their therapeutic utility in NAFLD management (Tables 1 and 2).

Accumulating evidence demonstrates that natural compounds and ingredients ameliorate NAFLD through gut-liver immune axis modulation. Polydatin is a naturally occurring stilbenoid compound, primarily derived from the Chinese medicinal plant Huzhang (*Polygonum cuspidatum* Sieb. et Zucc.). In TCM, it possesses the effects of promoting blood circulation to remove blood stasis, and draining dampness to relieve jaundice. Studies have confirmed that polydatin can alleviate diet-induced NASH and fibrosis in mice [163]. Furthermore, studies have demonstrated that Polydatin [163] and quercetin [164] suppress TLR4/NF-κB signaling in NASH models—the former by inhibiting CD68⁺ macrophage activation in MCD-diet mice, the latter via reversing endotoxin translocation from dysbiotic microbiota in HFD-fed mice, with quercetin concurrently inhibiting NLRP3 inflammasome and ER stress to normalize lipid metabolism genes. Gynostemma pentaphyllum polysaccharides (Jiaogulan polysaccharides) are a class of water-soluble polysaccharide ingredients extracted from Jiaogulan (*Gynostemma pentaphyllum* (Thunb.) Makino). Studies have confirmed that Gynostemma pentaphyllum polysaccharides exhibit antioxidant and immunomodulatory activities [165]. Bergamot flavonoids are a general term for a class of flavonoid ingredients extracted from the TCM plant Foshou (*Citrus medica* L. var. *sarcodactylis* Swingle). They possess the effects of soothing the liver and regulating qi, as well as harmonizing the stomach and relieving pain. Studies have found that bergamot flavonoids can significantly inhibit adipogenesis in NAFLD [166]. There is research indicating that gynostemma pentaphyllum polysaccharides [167] and a flavonoid-rich extract of bergamot juice [168] further expand this paradigm: both alleviate hepatic inflammation through TLR pathway inhibition (TLR2/NLRP3 and TLR4/MyD88/NF-κB/MAPK respectively), cytokine downregulation (TNF-α, IL-1β, IL-6), and gut barrier enhancement—notably reducing endotoxemia and improving intestinal permeability. Epigallocatechin-3-gallate (EGCG) is a major catechin compound, with its primary natural source being the leaves of Chashu (*Camellia sinensis* (L.) O. Ktze.). It is present in relatively high concentrations in green tea. In TCM, EGCG exhibits the effects of promoting fluid production to relieve thirst and aiding digestion to resolve food stagnation. Studies have demonstrated that it can regulate the intestinal immune microenvironment and reduce Th1 cell polarization, thereby ameliorating inflammatory bowel disease [169]. Research indicates that green tea polyphenol epigallocatechin-3-gallate (EGCG) directly targets intestinal immunity, as RNA-seq revealed its suppression of ileal IgA⁺ B-cell differentiation, LBP expression, and pro-inflammatory immune markers (CD11b⁺/CD11c⁺ dendritic cells, CD163⁺ macrophages, CD80/CD86 costimulators), thereby restoring mucosal immune homeostasis during NAFLD progression [170].

Dahuang Zhechong Pill (DHZCP), first documented in Zhang Zhongjing's Eastern Han Dynasty classic Jin Gui Yao Lue (Synopsis of the Golden Chamber), is composed of Tubiechong (*Eupolyphaga sinensis* Walker), Niuxi (*Achyranthes bidentata* Blume, *Rehmannia glutinosa* (Gaertn.) DC), Gancao (*Glycyrrhiza uralensis* Fisch. ex DC), Shuizhi (*Hirudo nipponica* Whitman), Baishao (*Paeonia lactiflora* Pall), Xingren (*Prunus armeniaca* L), Taoren (*Prunus persica* (L.) Batsch), Huangqin (*Scutellaria baicalensis* Georgi), Qicao (*Holotrichia oblita* Faldermann), Mangchong (*Tabanus bivittatus* Matsumura), and Dahuang (*Rheum palmatum* L). Its primary therapeutic actions are to invigorate blood circulation and resolve stasis, clear heat and moisten dryness, and nourish yin and enrich blood. DHZCP demonstrates significant efficacy in modulating immune function in liver system disorders, including NAFLD [171, 172]. A randomized controlled clinical trial revealed that Dahuang Zhechong Pill modulates immune function in NAFLD patients primarily by reducing the Th17/Treg cell ratio and decreasing serum levels of pro-inflammatory cytokines, including interleukin-17 (IL-17), high-sensitivity C-reactive protein (hs-CRP), and TNF-α [173].

Fuzi-Lizhong Decoction (FLZD), derived from the Song Dynasty's official compendium Taiping Huimin Heji Jufang (Formulary of the Peaceful Benevolent Dispensary), consists of Renshen (*Panax ginseng* C.A.Mey), Fuzi (*Aconitum carmichaelii* Debeaux), Gancao (*Glycyrrhiza uralensis* Fisch. ex DC), Baizhu (*Atractylodes macrocephala* Koidz), and Shengjiang (*Zingiber officinale* Roscoe). In treating NAFLD, FLZD significantly reduces pro-inflammatory cytokine expression and lowers serum lipid levels [174, 175]. Experimental studies further demonstrate that FLZD ameliorates hepatic inflammation and lipid accumulation in NAFLD models by inhibiting TLR4/MyD88/TRAF6 signaling pathway activation, consequently downregulating IL-2, IL-6, and TNF-α expression while reducing serum TC levels [176].

Qianggan Formula (QGF), developed by Shanghai University of Traditional Chinese Medicine based on TCM theoretical principles, consists of 16 herbal components: Yinchenhao (*Artemisia scoparia* Waldst. & Kit), Banlangen (*Isatis tinctoria* L), Danggui (*Angelica sinensis* (Oliv.) Diels), Baishao (*Paeonia lactiflora* Pall), Danshen (*Salvia miltiorrhiza* Bunge), Wenyujin (*Curcuma wenyujin* Y. H. Chen & C. Ling), Huangqi (*Astragalus membranaceus* (Fisch.) Bunge), Dangshen (*Codonopsis pilosula* (Franch.) Nannf), Zexie (*Alisma orientale* (Samuel.) Juz), Huangjing (*Polygonatum kingianum* Collett & Hemsl), Dihuang (*Rehmannia glutinosa* Libosch), Shanyao (*Dioscorea oppositifolia* L), Shanzha (*Crataegus pinnatifida* Bunge), Shenqu (Massa Medicata Fermentata), Qinjiao (*Gentiana macrophylla* Pall), and Gancao (*Glycyrrhiza uralensis* Fisch). Clinically used for chronic liver diseases, QGF treats NAFLD through modulation of bile acid metabolism and gut microbiota [177]. Furthermore, Zhu et al. demonstrated that QGF extract ameliorates NASH. Through microarray profiling of messenger RNA (mRNA), long non-coding RNA (lncRNA), and circular RNA (circRNA) expression validated by reverse transcription quantitative polymerase chain reaction (RT-qPCR), their research suggests that immunoregulatory ceRNA (competing endogenous RNA) networks involving lncRNA-circRNA interactions constitute potential therapeutic targets [178].

Dachaihu Decoction (DCHD), a classical formula originating from the Treatise on Febrile Diseases (Shang Han Lun), is composed of Beichaihu (*Bupleurum chinense* DC), Huangqin (*Scutellaria baicalensis* Georgi), Baishao (*Paeonia lactiflora* Pall), processed Banxia (*Pinellia ternata* (Thunb.) Breitenb), Shengjiang (fresh *Zingiber officinale* Roscoe rhizome), Suanzaoren (*Ziziphus jujuba* Mill), Dahuang (*Rheum palmatum* L. Rhubarb), and Zhishi (*Citrus* × *aurantium* L). Meta-analytical evidence confirms DCHD significantly improves liver function parameters and regulates dyslipidemia in NAFLD [179]. Notably, research on its core herb-pair combination (Bupleurum-Scutellaria-Paeonia decoction) demonstrates hepatoprotective effects in NAFLD rodent models through concurrent reductions in hepatic TG levels, pro-inflammatory cytokines including TNF-α and transforming growth factor-beta (TGF-β), key signaling mediators NF-κB and TLR4, and intestinal SIgA concentrations [180].

TCM-mediated regulation of the intestinal immune barrier centers on "balancing immune cell activity and modulating cytokine networks", encompassing both innate and adaptive immunity. Studies in this section confirm that TCM can regulate the gut-liver immune axis through multiple dimensions, providing diverse insights for the intervention of immune dysregulation in NAFLD.

### Bioavailability and administration routes of traditional Chinese medicine for intestinal barrier targeting in NAFLD

#### Overview of TCM bioavailability in NAFLD therapy

Bioavailability, defined as the fraction of an administered drug that reaches the systemic circulation and exerts biological effects, is a pivotal determinant of the therapeutic efficacy of TCM in NAFLD. TCM interventions for NAFLD, including bioactive ingredients and herbal compounds, face unique bioavailability challenges due to their chemical complexity, physiological barriers, and interactions with the gut-liver axis. Unlike synthetic drugs with well-characterized absorption profiles, TCM components often exhibit low aqueous solubility, poor membrane permeability, and extensive first-pass metabolism in the gut and liver, which collectively limit their bioavailability and clinical translation.

Critical analysis of existing literature reveals that most studies on TCM for NAFLD focus on mechanistic insights while neglecting quantitative and qualitative evaluations of bioavailability. For instance, lipophilic TCM ingredients such as curcumin (from Curcuma longa L.) and tanshinone IIA (from Salvia miltiorrhiza Bunge) have reported oral bioavailability of less than 1% in animal models, primarily due to poor solubility, rapid metabolism via cytochrome P450 enzymes, and efflux by intestinal transporters (e.g., P-glycoprotein) [181, 182]. Even hydrophilic components like polysaccharides (e.g., Poria cocos polysaccharides) face challenges in intestinal absorption, as their large molecular weights restrict paracellular and transcellular transport across the intestinal epithelium [183, 184].

The bioavailability of TCM is further influenced by the gut microbiota. While some TCM components are metabolized by gut bacteria into bioactive metabolites [185], others are degraded by pathogenic bacteria in NAFLD-related dysbiosis, reducing their effective concentration at target sites [186]. For example, chlorogenic acid, a phenolic acid with documented effects on gut microbiota modulation in NAFLD, is partially metabolized by Bifidobacterium and Lactobacillus into caffeic acid and ferulic acid [187]. However, in NAFLD model mice with depleted beneficial microbiota, this metabolic conversion is impaired, leading to reduced bioavailability of active metabolites [188].

### Impact of administration routes on TCM efficacy for intestinal barrier modulation

The choice of administration route directly affects TCM's delivery to the intestinal barrier and subsequent therapeutic effects in NAFLD. Oral administration, the most common route for TCM, allows direct interaction with the gut microbiota and intestinal epithelium but is limited by the aforementioned bioavailability issues. In contrast, alternative routes such as parenteral, nasal, or rectal administration may bypass intestinal barriers but compromise the gut-liver axis targeting that is central to TCM's mechanism of action in NAFLD.

### Oral administration

Oral delivery remains the preferred route for TCM targeting the intestinal barrier, as it enables direct contact with gut microbiota, bile acid pools, and epithelial cells. However, the efficacy of oral TCM is highly dependent on formulation strategies to improve bioavailability. Nanocarrier systems have been shown to enhance the solubility and intestinal absorption of curcumin and gypenosides, increasing their concentration in the gut and liver [189, 190]. Similarly, co-administration of absorption enhancers (e.g., piperine from Piper nigrum) can inhibit P-glycoprotein efflux and cytochrome P450 metabolism, improving the bioavailability of lipophilic TCM ingredients [191]. Notably, the gastrointestinal environment in NAFLD patients further modulates oral TCM bioavailability. For instance, increased intestinal permeability in NAFLD may enhance the absorption of some TCM components but also lead to uncontrolled translocation of endotoxins, which can interfere with TCM's anti-inflammatory effects [192].

### Alternative administration routes

Alternative administration routes have been explored to overcome the limitations of oral delivery, although their application in TCM for NAFLD is relatively limited. Intravenous injection bypasses intestinal absorption barriers and ensures maximum bioavailability but lacks direct interaction with the gut microbiota and intestinal barrier, weakening the gut-liver axis modulation that is critical for TCM's efficacy [193]. Nasal and rectal administration offer potential advantages for TCM targeting the intestinal barrier. Nasal delivery bypasses first-pass metabolism and allows direct transport to the systemic circulation and liver via the olfactory nerve, while rectal administration avoids gastric acid degradation and enhances local absorption in the colon. However, these routes are less patient-compliant and require specialized formulations that are not widely used in TCM clinical practice [194, 195].

### Targeted delivery systems

Targeted delivery systems represent a promising strategy to improve TCM bioavailability and intestinal barrier targeting. Research has shown that oral nano-medicine can enhance the function of the intestinal barrier, regulate the intestinal microbiota and microbial metabolites [196]. Ligand-modified nanocarriers can facilitate the delivery of macromolecular drugs across the intestinal mucosa [197]. Al-Okbi et al. developed a nanoemulsion of basil essential oil. The results showed that this nanoemulsion could reduce the progression of NASH and had an advantage in reducing the ratio of Firmicutes to Bacteroidetes in the intestinal microbiota (F/B) [198]. Berberine (BBR), a botanical medicine, shows therapeutic efficacy in patients with metabolic diseases. A colon-specific delivery system, BBR-CS/PT-NP, is investigated by the assembly of pH/gut microflora dual stimuli-responsive nanoparticles for enhancing the interaction between BBR and gut microbiota. In high-fat diet-induced obese hamsters, intervention with BBR-CS/PT-NP significantly alleviated hepatic steatosis, and its therapeutic efficacy was superior to that of pure BBR does. The results provide a novel proof-of-concept for drug delivery targeting gut microbiota to ameliorate metabolic disorders [199].

### Critical challenges and future directions

Despite the accumulating evidence supporting the therapeutic potential of TCM in regulating the intestinal barrier for NAFLD management, several critical challenges remain to be addressed. These challenges primarily stem from the unclear causal relationship between TCM-mediated intestinal barrier regulation and NAFLD improvement, the inherent chemical complexity of TCM and bioavailability evaluation, unaddressed herb-drug interactions (HDIs), and the lack of standardized research methodologies.

### Correlational and causal relationships in TCM-mediated intestinal barrier regulation

A critical challenge in understanding how TCM alleviates NAFLD is distinguishing between correlation and causation in its effects on the intestinal barrier. Much of the current evidence is correlational, documenting synchronous improvements—such as between chlorogenic acid's modulation of Bifidobacterium and reduced liver fat, or between Poria cocos polysaccharides' upregulation of Occludin and mitigated liver injury. While these associations are valuable for generating hypotheses, they cannot confirm that intestinal barrier repair is the direct, necessary mechanism of action, as TCM components may also act directly on the liver. Establishing causality requires rigorous validation proving that TCM's efficacy depends on specific intestinal barrier changes.

To elucidate the causal mechanisms by which TCM alleviates NAFLD, future research must move beyond correlational observations and adopt targeted intervention strategies that align with TCM's multi-target characteristics. For the intestinal mechanical barrier hypothesis, gene silencing techniques could be used to specifically knock down key tight junction proteins (e.g., Occludin, ZO-1), or chemical agents could be applied to disrupt barrier integrity, thereby testing whether barrier restoration is a necessary prerequisite for TCM's efficacy. For the gut microbiota hypothesis, broad-spectrum antibiotics could be employed to deplete the microbiota to verify therapeutic dependency, while fecal microbiota transplantation (FMT) experiments could confirm the sufficiency of the microbiota as a mediator. For the bile acid or immune regulation hypotheses, specific receptor antagonists or gene-knockout animal models could be utilized to block targeted pathways and observe whether the therapeutic effects are reversed. This layered and targeted verification framework is expected to systematically dissect and establish the precise causal roles of various intestinal barrier components within TCM's complex mechanism of action against NAFLD.

### Complexity of TCM chemical composition and bioavailability evaluation

Despite the progress in understanding TCM bioavailability and administration routes, several critical challenges remain. The inherent chemical complexity of traditional Chinese medicine, characterized by multi-component formulas containing a diverse array of bioactive compounds such as flavonoids, alkaloids, polysaccharides, and terpenoids, presents a significant challenge for accurately quantifying the bioavailability of individual constituents and their metabolites [200]. Unlike synthetic drugs with well-characterized absorption, distribution, metabolism, and excretion (ADME) profiles, TCM components often exhibit overlapping pharmacokinetic behaviors and potential synergistic or antagonistic interactions [201]. Most existing studies focus on single TCM ingredients or crude extracts, neglecting qualitative and quantitative evaluations of multi-component interactions, which limits the establishment of precise dose–response relationships for clinical application.

Furthermore, significant individual differences exist in NAFLD-related pathophysiological alterations such as intestinal dysbiosis, increased intestinal permeability, and hepatic steatosis, leading to heterogeneity in the bioavailability and therapeutic responses of TCM. This variability is particularly pronounced in patients with severe intestinal dysbiosis [202]. An individualized medical strategy that adjusts TCM formulas based on intestinal flora profiles or metabolic phenotypes may address this issue, but it requires large-scale clinical validation and standardized evaluation tools.

Future research should prioritize the development of standardized methods to evaluate TCM bioavailability in NAFLD models and clinical trials. This includes the use of advanced analytical techniques (e.g., liquid chromatography-mass spectrometry) to quantify TCM components and their metabolites in plasma, gut, and liver tissues, as well as the integration of pharmacokinetic and pharmacodynamic data to establish dose–response relationships. Additionally, preclinical and clinical studies should compare the efficacy of different administration routes and formulations, focusing on their impact on intestinal barrier function, gut microbiota composition, and NAFLD pathology. Another important direction is the identification of bioavailability-enhancing strategies specific to TCM. This includes the optimization of formulation techniques and the discovery of natural absorption enhancers from TCM herbs that can improve the solubility and permeability of TCM components without causing adverse effects. Furthermore, understanding the interplay between TCM bioavailability and gut microbiota metabolism may lead to the development of combination therapies (e.g., TCM plus probiotics) that synergistically enhance intestinal barrier function and NAFLD treatment outcomes.

### Unaddressed HDIs in clinical NAFLD management

A key knowledge gap in current research is the insufficient investigation into HDIs. HDIs are a growing concern in modern healthcare, with almost 70% of individuals using herbal remedies alongside conventional pharmaceuticals. Herbal molecules can interact with medicines via pharmacodynamic pathways, leading to antagonistic, combined, and synergistic effects [203]. These interactions can have either beneficial or adverse consequences, and the concentration of a medicine in a certain tissue may change due to these interactions. Given that patients with NAFLD often have comorbidities such as type 2 diabetes mellitus, hypertension, and dyslipidemia that require long-term Western medication [204], HDIs represent a core consideration for the safe application of TCM in NAFLD management. NAFLD patients frequently use hypoglycemic agents, lipid-lowering drugs, and anti-inflammatory medications, and the combination of TCM with these Western drugs may affect their efficacy or safety through several mechanisms: by modulating cytochrome P450 drug-metabolizing enzymes; by competing for intestinal or hepatic transporters like P-glycoprotein; or by altering gut microbiota responsible for drug biotransformation [205]. Current HDIs research remains limited by its focus on single ingredients rather than the compound formulations used in clinical practice, its frequent use of in vitro or healthy animal models that fail to recapitulate NAFLD-specific pathophysiology, and a notable absence of clinical validation.

Clinically, with the increasing prevalence of combined TCM and Western Medicine therapy, HIDs and the occurrence of adverse reactions have become critical factors requiring careful consideration prior to clinical combination treatment. Owing to the unclear chemical composition of most TCM, their pharmacokinetic profiles and mechanisms of action remain largely undefined. When TCM are co-administered with Western Medicines, the absorption, distribution, metabolism, and excretion processes of active components in Western Medicines may undergo significant alterations. Improper combination not only modifies therapeutic efficacy but also potentially induces toxic side effects; in contrast, rational combination can enhance efficacy while avoiding adverse reactions. Therefore, combined medication should be implemented based on the pharmacokinetic characteristics of TCM and Western Medicines. By monitoring changes in Western Medicine efficacy and pharmacokinetic profiles following TCM-Western Medicine combination, it is feasible to analyze the pharmacology, properties, and action patterns of TCM, as well as to elucidate the mechanisms underlying drug interactions. This approach provides evidence for the rational use of TCM-Western Medicine combinations and promotes safe clinical medication practice.

### Standardization of preclinical and clinical research methods

A critical bottleneck hindering the translation of TCM research into clinical practice and its international recognition lies in the prevalent lack of standardization in preclinical and clinical study designs. At the preclinical stage, inconsistencies in TCM extraction protocols—such as solvent type, extraction temperature and duration, and concentration ratios—often lead to significant variations in the concentrations of bioactive components. This not only reduces the reproducibility of experimental results but also impedes the establishment of clear dose–effect relationships between TCM components and therapeutic outcomes. More critically, human NAFLD patients frequently present with comorbidities including insulin resistance, type 2 diabetes mellitus, and gut microbiota dysbiosis, while common animal models used for investigating TCM interventions in NAFLD fail to fully recapitulate the multifactorial pathophysiological characteristics of human NAFLD. This "model-clinic disconnect" severely limits the translational value of preclinical research findings.

At the clinical level, most RCTs evaluating TCM for NAFLD suffer from small sample sizes, which not only reduce statistical power but also increase the risk of false-positive or false-negative results. Furthermore, outcome measures vary substantially across studies, and long-term safety data are generally lacking, making it impossible to capture potential adverse reactions associated with prolonged TCM use. This gap in safety evidence undermines the evidential basis for the efficacy and safety of TCM.

Therefore, in the future, unified standard operating procedures for TCM extraction should be established, and methods for detecting bioactive components should be standardized to ensure the reproducibility of experiments. Animal models that more closely mimic the pathophysiological characteristics of human NAFLD ought to be developed to enhance the translational value of preclinical research. In clinical research, large-sample, multicenter RCTs should be conducted, with unified outcome measures and extended follow-up periods, so as to facilitate the clinical promotion and international recognition of TCM.

## Conclusion

This review synthesizes current evidence on intestinal barrier impairment in NAFLD, elucidates the multifaceted mechanisms by which TCM—encompassing compound formulations, active ingredients, and established herbal prescriptions—exerts therapeutic effects, and discusses the bioavailability and optimal administration routes of TCM targeting the intestinal barrier in NAFLD, as well as the existing problems and challenges. Further exploration of the interplay between TCM and the intestinal barrier is essential to enrich TCM theoretical frameworks, drive the development of novel intestinal barrier-targeted TCM preparations, and provide innovative clinical strategies for NAFLD.

## Data Availability

No datasets were generated or analysed during the current study.

## Abbreviations

AJs: Adherens junctions
AST: Aspartate aminotransferase
Bas: Bile acids
CA: Cholic acid
CDCA: Chenodeoxycholic acid
CHOL: Cholesterol
circRNA: Circular RNA
CVD: Cardiovascular diseases
CYP7A1: Cholesterol 7α-hydroxylase
CYP8B1: Cholesterol 12α-hydroxylase
DAMPs: Damage-associated molecular patterns
EGCG: Epigallocatechin-3-gallate
FXR: Farnesoid X Receptor
GALT: Gut-associated lymphoid tissue
GPBAR1/TGR5: G protein-coupled bile acid receptor 1
HDL-C: High-density lipoprotein cholesterol
HFD: High-fat diet
hs-CRP: High-sensitivity C-reactive protein
IBD: Inflammatory bowel disease
IELs: Intraepithelial lymphocytes
IL-17: Interleukin-17
LBP: Lipopolysaccharide-binding protein
LPS: Lipopolysaccharide
lncRNA: Long non-coding RNA
LPLs: Lamina propria lymphocytes
MCD: Methionine-choline deficient
MAPK: Mitogen-activated protein kinase
MAFLD: Metabolic-associated Fatty Liver Disease
mRNA: Messenger RNA
MyD88: Myeloid differentiation factor 88
NAFLD: Non-alcoholic fatty liver disease
NASH: Nonalcoholic steatohepatitis
NF-κB: Nuclear factor-κB
PAMPs: Pathogen-associated molecular patterns
PPARα: Peroxisome proliferator-activated receptor α
PXR: Pregnane X receptor
qPCR: Quantitative polymerase chain reaction
RCTs: Randomized controlled trials
RT-qPCR: Reverse transcription quantitative polymerase chain reaction
SCFAs: Short-chain fatty acids
SHP: Small heterodimer partner
SIgA: Secretory immunoglobulin A
SIRT6: Sirtuin 6
SREBP1c: Sterol regulatory element-binding protein 1c
TG: Triglycerides
T1DM: Type 1 diabetes mellitus
TCM: Traditional Chinese Medicine
TLR4: Toll-like receptor-4
TLRs: Toll-like receptors
TJs: Tight junctions
TGF-β: Transforming growth factor-beta
TNF-α: Tumor necrosis factor-α
HDIs: Herb-drug interactions
